# Identification of novel SNPs associated with coronary artery disease and birth weight using a pleiotropic cFDR method

**DOI:** 10.18632/aging.202322

**Published:** 2020-12-19

**Authors:** Xinrui Wu, Xu Lin, Qi Li, Zun Wang, Na Zhang, Mengyuan Tian, Xiaolei Wang, Hongwen Deng, Hongzhuan Tan

**Affiliations:** 1 Department of Epidemiology and Health Statistics, Xiangya School of Public Health, Central South University, Changsha 410078, China; 2 Department of Endocrinology and Metabolism, The Third Affiliated Hospital of Southern Medical University, Guangzhou 510630, China; 3 Xiangxi Center for Disease Prevention and Control, Jishou 416000, China; 4 Xiangya Nursing School, Central South University, Changsha 410013, China; 5 School of Basic Medical Science, Central South University, Changsha 410013, China; 6 Tulane Center for Biomedical Informatics and Genomics, School of Medicine, Tulane University, New Orleans, LA 70112, USA

**Keywords:** coronary artery disease, birth weight, conditional FDR, pleiotropy, Mendelian randomization

## Abstract

Objectives: Clinical and epidemiological findings indicate an association between coronary artery disease (CAD) and low birth weight (BW). However, the mechanisms underlying this relationship are largely unknown. Here, we aimed to identify novel single-nucleotide polymorphisms (SNPs) associated with CAD, BW, and their shared pleiotropic loci, and to detect the potential causal relationship between CAD and BW.

Methods: We first applied a genetic pleiotropic conditional false discovery rate (cFDR) method to two independent genome-wide association studies (GWAS) summary statistics of CAD and BW to estimate the pleiotropic enrichment between them. Then, bi-directional Mendelian randomization (MR) analyses were performed to clarify the causal association between these two traits.

Results: By incorporating related traits into a conditional analysis framework, we observed the significant pleiotropic enrichment between CAD and BW. By applying the cFDR level of 0.05, 109 variants were detected for CAD, 203 for BW, and 26 pleiotropic variants for both traits. We identified 11 CAD- and/or BW-associated SNPs that showed more than three of the metabolic quantitative trait loci (metaQTL), protein QTL (pQTL), methylation QTL (meQTL), or expression QTL (eQTL) effects. The pleiotropic SNP rs10774625, located at *ATXN2,* showed metaQTL, pQTL, meQTL, and eQTL effects simultaneously. Using the bi-directional MR approach, we found a negative association from BW to CAD (odds ratio [OR] = 0.68, 95% confidence interval [CI]: 0.59 to 0.80, *p* = 1.57× 10^-6^).

Conclusion: We identified several pleiotropic loci between CAD and BW by leveraging GWAS results of related phenotypes and identified a potential causal relationship from BW to CAD. Our findings provide novel insights into the shared biological mechanisms and overlapping genetic heritability between CAD and BW.

## INTRODUCTION

Coronary artery disease (CAD) is characterized by the narrowing or obstruction of the coronary arteries, which can lead to chest pain, arrhythmia, heart failure, and even permanent heart damage [1]. In 2017, over 485 million people suffered from CAD, resulting in 17.8 million deaths [2, 3], making this disease the leading cause of morbidity and mortality worldwide [4].

Numerous studies have shown that early life experiences, including low birth weight (BW), may increase the risk of cardiovascular diseases [5–7]. Thus, the World Health Organization has classified low BW as a risk factor for CAD later in life [8]. However, the prevalence of CAD does not decrease with higher BW accompanied by improved living conditions [9]. In addition, many randomized controlled trials designed to improve BW revealed different results [10, 11], leaving the relationship between BW and CAD unclear.

CAD and BW are highly influenced by multiple genetic factors with heritability estimates over 30–60% [12] and 30–50% [13], respectively. With the development of genome-wide association studies (GWAS), more than 230 CAD-associated [14–19] and 80 BW-associated loci [20–22] have been detected. These loci describe only a small part of the genetic contribution [23, 24], leaving a large proportion of “missing heritability” unexplained [25]. Pleiotropy occurs when one gene or variant affects multiple phenotypes [26]. Among the human genome, more than 17% of genes and 5% of single-nucleotide polymorphisms (SNPs) show pleiotropic effects [27]. Considering the potential causal relationship, large genetic determination, pleiotropic effect, and missing heritability between CAD and BW, it is necessary to illuminate biological mechanisms and uncover novel associated genetic variants for both traits.

By leveraging the pleiotropic effect in related traits, a conditional false discovery rate (cFDR) method was developed without additional subjects recruitment [28]. This approach is cost-effective and could improve the identification of novel genetic loci underlying missing heritability, thereby elucidating genetic mechanisms associated with multiple phenotypes [29–32]. Furthermore, Mendelian randomization (MR) is an approach to investigate the potential causality between exposure and outcome using genetic instrumental variables [33]. As genetic variants are randomly distributed among the population and are generally independent of confounders, such analysis may reduce confounding bias and eliminate potential reversed causal relationship [34].

In this study, we applied cFDR and bi-directional MR analyses to two large and independent GWAS datasets aiming to 1) identify additional novel loci and the genetic pleiotropy of CAD and BW, and 2) estimate the causality between CAD and BW. Therefore, we can improve SNP detection, and clarify the shared mechanic relationship and overlapping genetic heritability between these two traits better.

## RESULTS

### Pleiotropic enrichment estimation

**Figure 1:**
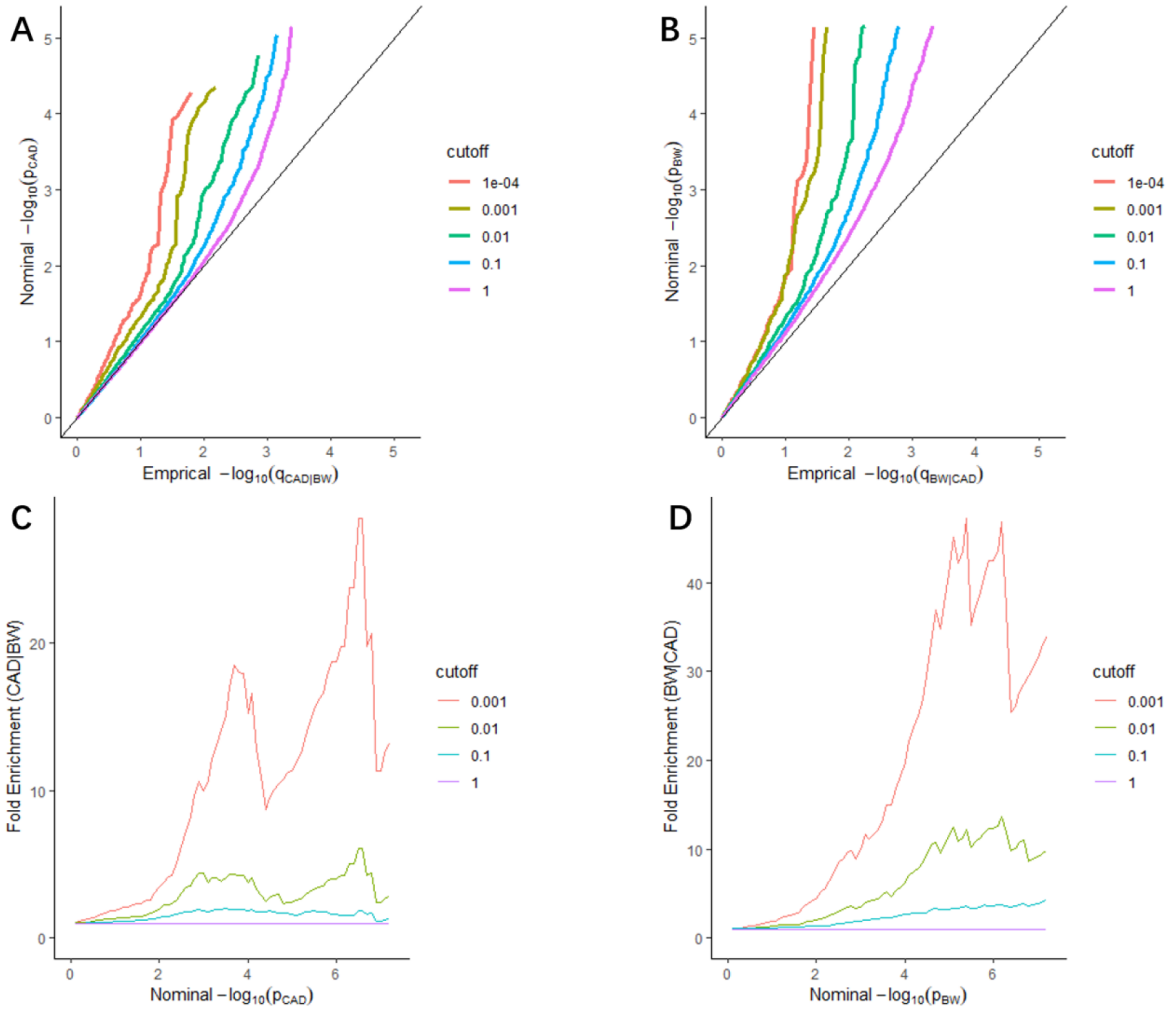
**Stratified Q-Q plots and fold-enrichment plots.** Stratified Q-Q plots of nominal vs. empirical -log10(*p*) values in principal trait below the standard GWAS threshold of *p* ≤ 5 × 10^−8^ as a function of the significance of the association with conditional trait at the level of *p* ≤ 1, *p* ≤ 0.1, *p* ≤ 0.01, *p* ≤ 0.001, and *p* ≤ 0.0001, respectively. (**A**) CAD as a function of the significance of the association with BW, and (**B**) BW as a function of the significance of the association with CAD. Fold-enrichment plots of enrichment vs nominal -log10(*p*) values (corrected for inflation) corresponding to levels of *p* ≤ 1, *p* ≤ 0.1, *p* ≤ 0.01, ≤ 0.001, respectively in (**C**) CAD below the standard GWAS threshold of *p* ≤ 5 × 10^−8^ as a function of significance of the association with BW; and in (**D**) BW below the standard GWAS threshold of *p* ≤ 5 × 10^−8^ as a function of significance with CAD. Dashed lines indicate the null-hypothesis.

We found leftward separations between each line (including the null line) in the stratified quantile-quantile (Q-Q) plots, which indicated the pleiotropy of CAD conditional on BW (Figure 1A), as well as BW conditional on CAD (Figure 1B). As shown in fold-enrichment plots (Figure 1C, 1D), distinct upward shifts from the baseline demonstrated a strong pleiotropic enrichment between BW and CAD. We observed the most notable pleiotropy with an enrichment fold greater than 40 in BW conditional on CAD.

Furthermore, the stratified Q-Q plots for CAD conditional on autism spectrum disorder (ASD) (Supplementary Figure 1A), and BW conditional on ASD (Supplementary Figure 1C) all showed no enrichment and vice versa (Supplementary Figure 1B, 1D), which can be the negative controls.

### CAD-associated SNPs identified by cFDR

**Figure 2:**
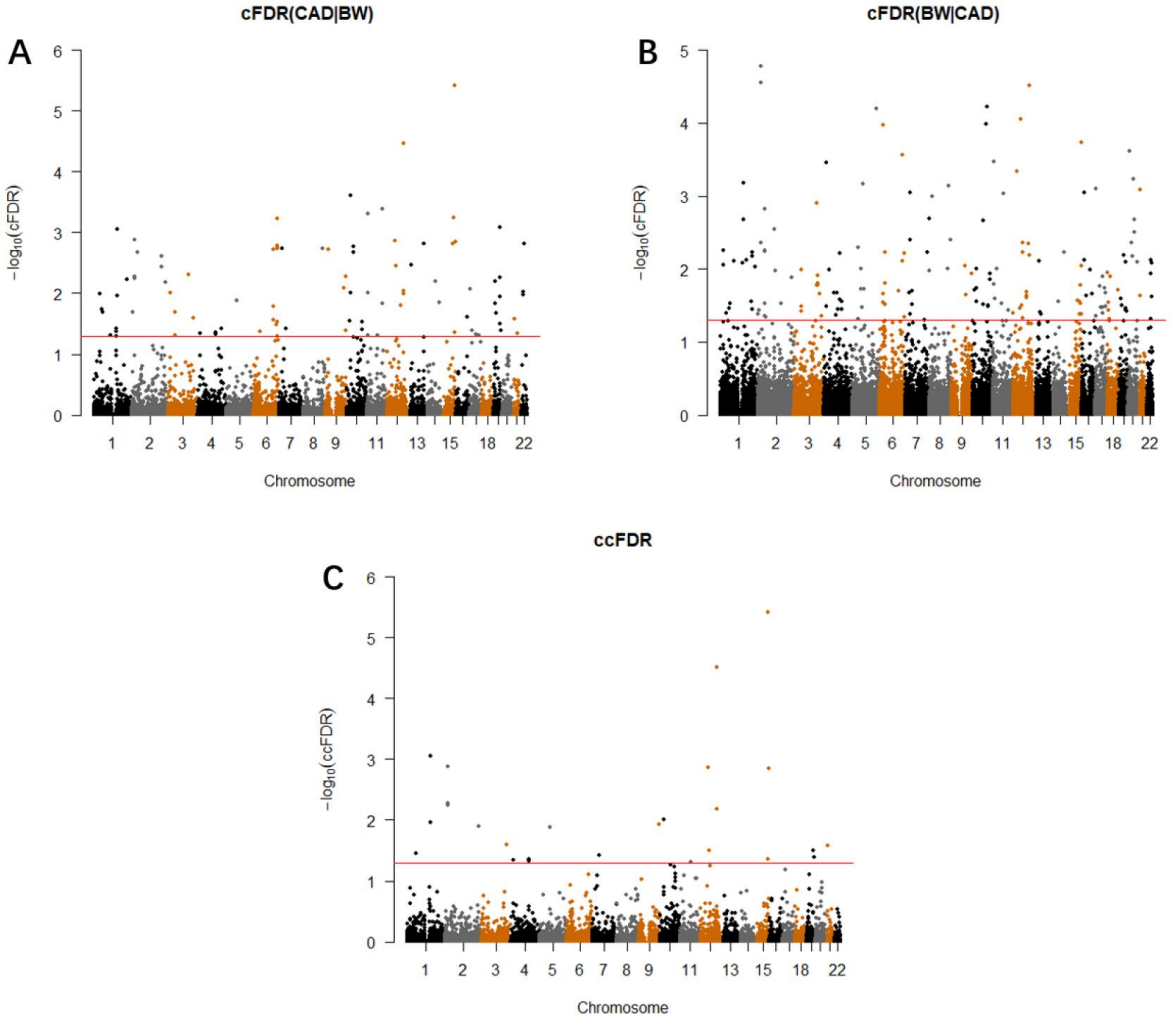
**Conditional Manhattan plot.** SNPs with -log10(cFDR) ≥ 1.3 (cFDR ≤ 0.05) for (**A**) CAD given BW (CAD|BW) and (**B**) BW given CAD (BW|CAD), or (**C**) -log10(ccFDR) ≥ 1.3 (ccFDR ≤ 0.05) are shown above the red line.

Conditional on BW, we identified 109 significant SNPs (cFDR ≤ 0.05) associated with CAD variation, which were located on 20 different chromosomes (1–17, 19, 21–22), annotated to 98 genes (Supplementary Table 1 and Figure 2A). We validated 22 SNPs that were statistically significant in the former CAD GWAS datasets [14–19]. Additionally, six SNPs associated with cardiovascular function were also validated in our research [35–38]. Excluding 26 SNPs that showed high linkage disequilibrium (LD) (r2 > 0.6) with the previous CAD-related loci, the remaining 55 SNPs were potentially novel to CAD (Supplementary Table 2). Using validation datasets, we found 111 significant SNPs for CAD, 73 of which (65.8%) were also significant in the original cFDR research (Supplementary Table 3).

We detected 16 SNPs associated with various metabolites (Supplementary Table 7), such as kynurenine, C18:1 sphingomyelin, and cholesterol, which affected the pathogenesis of CAD. Seven SNPs were associated with different proteins, and 27 SNPs showed significant metabolic quantitative trait locus (metaQTL) effects in the human serum. Notably, three novel SNPs, rs11244035, rs3811417, and rs624249, showed more than three metaQTL, protein QTL (pQTL), methylation QTL (meQTL), or expression QTL (eQTL) effects simultaneously (Table 1).

**Table 1:** Conjunction cFDR for 26 pleiotropic SNPs in CAD and BW (ccFDR ≤ 0.05).

**SNP**	**Chr**	**Pos**	**Alt**	**Gene**	**Annotation**	**metaQTL/pQTL/meQTL/eQTL**	**SNP Type**	**Gene Type**	**cFDR_CAD**	**cFDR_BW**	**ccFDR**
rs10774625	12	111472415	A/T	*ATXN2*	intronic	metaQTL/pQTL/meQTL/eQTL(3 hits)	CAD	CAD	1.48E-08	3.06E-05	3.06E-05
rs11066301	12	112433568	A/T	*PTPN11*	intronic	metaQTL/meQTL/eQTL(1 hit)	CAD	CAD	3.45E-05	6.50E-03	6.50E-03
rs11172113	12	57133500	T/A	*LRP1*	intronic	metaQTL/meQTL/eQTL(4 hits)	Novel	Novel	3.56E-03	3.18E-02	3.18E-02
rs11206803	1	56411837	C/G	*AC119674.2*	intronic	meQTL	Novel	Novel	2.02E-02	3.51E-02	3.51E-02
rs12148530	15	96542056	T/A	*7SK*	intergenic		Novel	Novel	4.40E-02	1.64E-02	4.40E-02
rs12306172	12	54145221	G/C	*SMUG1*	intronic	eQTL(7 hits)	Novel	Novel	1.37E-03	8.89E-05	1.37E-03
rs13035774	2	24135782	C/G	*FAM228B*	intronic	meQTL/eQTL(29 hits)	Novel	Novel	5.73E-03	4.35E-03	5.73E-03
rs1319869	15	98669256	G/C	*IGF1R*	intronic		Novel	BW	1.41E-03	1.85E-04	1.41E-03
rs1480933	4	119512093	C/G	*PDE5A*	intronic	pQTL/eQTL(17 hits)	Novel	Novel	4.43E-02	3.52E-02	4.43E-02
rs1861044	4	15537875	A/T	*CC2D2A*	intronic	pQTL	Novel	Novel	4.54E-02	3.25E-02	4.54E-02
rs2268310	7	44637499	C/G	*OGDH*	intronic	meQTL	Novel	Novel	3.85E-02	3.10E-02	3.85E-02
rs2339940	2	24028917	G/C	*MFSD2B*	intronic	eQTL(22 hits)	Novel	Novel	1.34E-03	1.67E-05	1.34E-03
rs3756668	5	68300260	G/C	*PIK3R1*	3'-UTR		Novel	Novel	1.32E-02	6.91E-04	1.32E-02
rs4233701	2	23706216	G/C	*KLHL29*	intronic	eQTL(15 hits)	Novel	Novel	5.26E-03	2.81E-05	5.26E-03
rs4643791	4	119344464	G/C	*FABP2*	intergenic	eQTL(21 hits)	Novel	Novel	4.76E-02	2.75E-02	4.76E-02
rs502467	3	172009573	T/A	*FNDC3B*	intergenic		Novel	Novel	2.58E-02	2.22E-02	2.58E-02
rs611003	11	69630516	C/G	*CCND1*	intergenic		Novel	Novel	4.98E-02	9.20E-04	4.98E-02
rs630014	9	133274306	A/T	*ABO*	intronic	metaQTL/meQTL/eQTL(9 hits)	Novel	CAD	5.22E-03	1.16E-02	1.16E-02
rs6673081	1	155017119	T/A	*ZBTB7B*	3'-UTR	eQTL(8 hits)	Novel	BW	8.98E-04	4.66E-08	8.98E-04
rs670950	19	43777410	T/A	*KCNN4*	intronic	eQTL(1 hit)	Novel	Novel	3.15E-02	7.91E-03	3.15E-02
rs6713510	2	226169783	G/C	*LOC646736*	intronic		CAD	CAD	6.65E-03	1.29E-02	1.29E-02
rs8039305	15	90879313	T/A	*FURIN*	intronic	meQTL/eQTL(27 hits)	Novel	CAD	3.77E-06	1.13E-06	3.77E-06
rs8105944	19	51047598	C/G	*KLK13*	intergenic		Novel	Novel	4.04E-02	3.81E-02	4.04E-02
rs821551	1	155718789	C/G	*DAP3*	intronic	meQTL/eQTL(50 hits)	Novel	Novel	1.08E-02	6.67E-04	1.08E-02
rs866919	10	30224354	C/G	*RP11*	intergenic	eQTL(1 hit)	Novel	Novel	9.93E-03	9.87E-03	9.93E-03
rs965098	21	15185306	G/C	*JCAD*	intergenic		Novel	Novel	2.64E-02	2.35E-02	2.64E-02

Abbreviations: Chr, chromosome; Pos, chromosomal position (GRCh38/hg38); metaQTL, metabolic quantitative trait locus; pQTL, protein quantitative trait locus; meQTL, methylation quantitative trait locus; eQTL, expression quantitative trait locus; CAD, coronary artery disease; BW, birth weight; cFDR, conditional false discovery rate; ccFDR, conjunctional conditional false discovery rate. The allele was exhibited as reference allele/alter allele; SNP type and gene type means whether identified SNPs and genes have been reported in previous GWAS or in previous related cFDR studies.

### BW–associated SNPs identified by cFDR

Conditional on CAD, we identified 203 significant SNPs (cFDR ≤ 0.05) associated with BW variation, which were located on 22 chromosomes (1–22), annotated to 179 genes (Supplementary Table 4 and Figure 2B). We validated 27 SNPs that were statistically significant in the former BW GWAS datasets [20–22, 39], although 19 of the remaining 176 SNPs showed high LD (r2 > 0.6) with the previous BW-related loci (Supplementary Table 5). Using validation datasets, we found 229 significant SNPs for BW, 182 of which (79.5%) were also significant in the original cFDR research (Supplementary Table 6).

We detected 26 SNPs associated with various metabolites (Supplementary Table 7), five were associated with different proteins, and 31 showed significant meQTL effects in the human serum. In particular, four novel SNPs, rs143384, rs4875812, rs6700896, and rs8108865, showed more than three metaQTL, pQTL, meQTL, or eQTL effects simultaneously (Table 1).

### Potentially pleiotropic SNPs identified using conjunction cFDR (ccFDR)

We calculated the ccFDR value and constructed the conjunction Manhattan plot to explore the pleiotropic loci between CAD and BW. (Figure 2C). Precisely 26 potentially pleiotropic loci that reached a significance threshold at ccFDR ≤ 0.05 were mapped to 13 chromosomes and annotated to 26 different genes. We validated three SNPs that were statistically significant in the original GWAS and CAD-related study, nine loci were also found to be related to other phenotypes (Supplementary Table 8). Using validation datasets, we found 17 pleiotropic SNPs for both traits, 12 of which (70.5%) were also pleiotropic loci in the original ccFDR research (Supplementary Table 9). We then detected 18 pleiotropic SNPs that showed more than one of the metaQTL, pQTL, meQTL, or eQTL effects. Particularly, rs10774625 showed all QTL effects simultaneously (Table 2).

**Table 2:** Functional annotation for 11 SNPs showing significant effects in metaQTL, pQTL, meQTL, and eQTL.

**SNP**	**GENCODE genes**	**Traits**	**metaQTL**	**pQTL**	**meQTL (*P*)**	**eQTL Hits**	**Promoter histone marks**	**Enhancer histone marks**	**DNAse**	**Proteins bound**	**Motifs changed**
rs10774625	*ATXN2*	Pleiotropic	9 hits	B2M	4.10E-16	3 hits					9 altered motifs
rs11066301	*PTPN11*	Pleiotropic	2 hits		6.48E-12	1 hit		BLD			6 altered motifs
rs11172113	*LRP1*	Pleiotropic	SM C18:1		1.43E-07	4 hits	8 tissues	15 tissues	17 tissues	FOXA1	AP-2, Hic1, PU.1
rs630014	*ABO*	Pleiotropic	2 hits		4.82E-09	9 hits	4 tissues	GI, MUS	ESC,GI		Gm397, RP58
rs11244035	*OBP2B*	CAD		8 hits	1.37E-05	6 hits					Ik-1, Ik-2, NERF1a
rs3811417	*RORC*	CAD	nonanoylcarnitine		5.46E-06	2 hits	5 tissues	12 tissues	CRVX		Arnt, Mxi1, Myc
rs624249	*SLC22A2*	CAD	X-12798		9.90E-05	3 hits		4 tissues			
rs143384	*GDF5*	BW		CPN1	1.27E-07	47 hits	9 tissues	13 tissues	16 tissues		Ascl2
rs4875812	*MIR596*	BW	deoxycholate		3.11E-12	3 hits		4 tissues			9 altered motifs
rs6700896	*LEPR*	BW		LEPR	3.16E-07	1 hit		LIV	SKIN,SKIN	CTCF	GR, Myf, TCF12
rs8108865	*FCHO1*	BW	HWESASXX		1.27E-28	1 hit		BRN, BLD			NF-Y, NF-kappaB, Pou2f2

Abbreviations: metaQTL, metabolic quantitative trait locus; pQTL, protein quantitative trait locus; meQTL, methylation quantitative trait locus; eQTL, expression quantitative trait locus; DNAse, deoxyribonuclease; SM C18:1, C18:1 sphingomyelin; B2M, beta-2-microglobulin.

### Causality between BW and CAD

**Figure 3:**
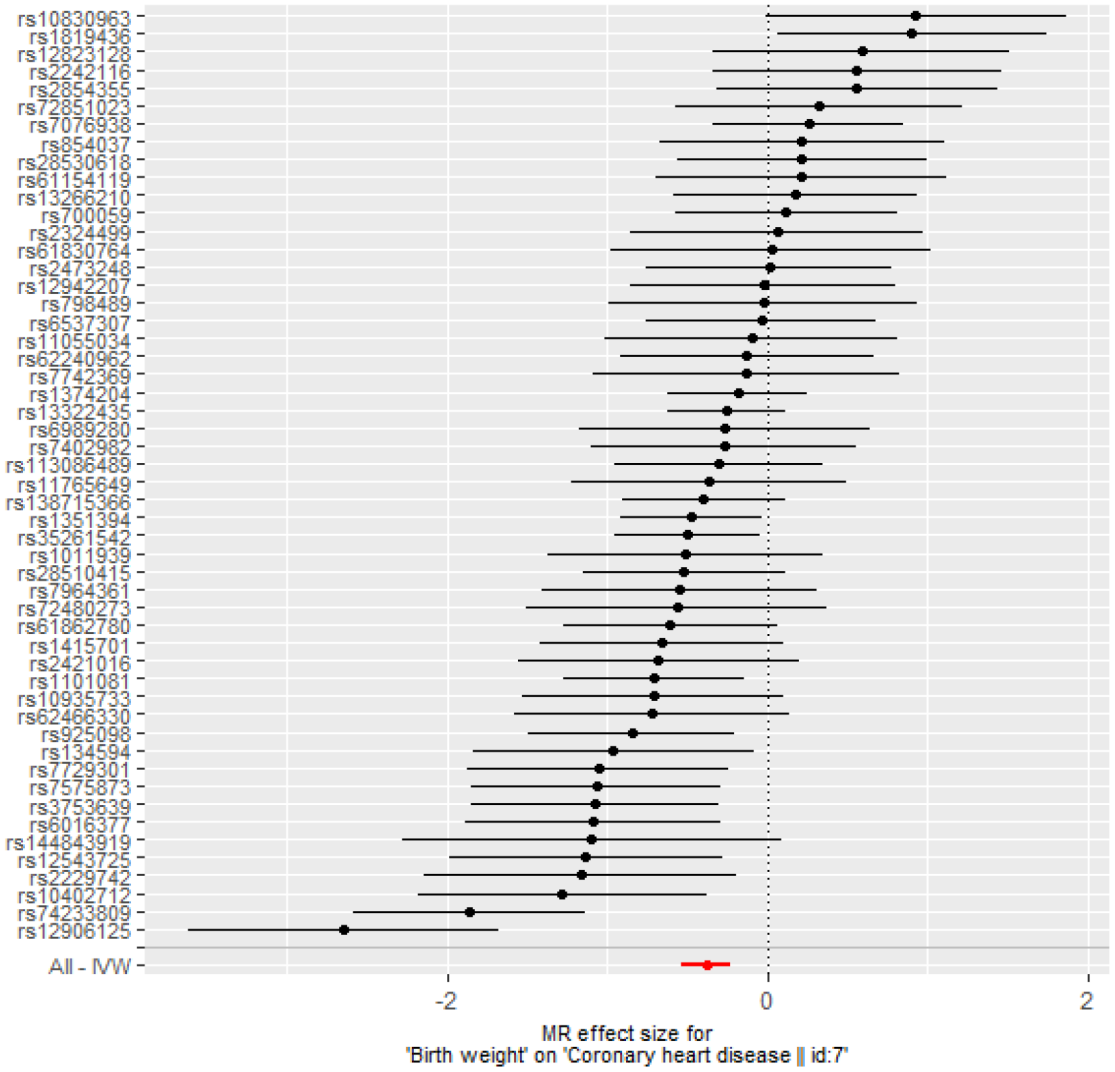
**Forest plot of MR estimates BW on CAD.** The estimated causal effect of BW on CAD was expressed by IVW (OR= 0.68, 95% CI: 0.59 to 0.80, *p* = 1.57× 10^-6^).

After instrument selection, LD clumping, variant extraction, and harmonization, 52 BW-CAD SNP pairs were selected when choosing BW as exposure (Supplementary Table 10). The MR-Egger regression test result (intercept: -0.0025, 95% confidence interval [CI]: -0.015 to 0.014, *p* = 0.973) suggested that there was no genetic confounding due to horizontal pleiotropy. The null-pleiotropy result was also confirmed using scatter plots and funnel plots (Supplementary Figures 2, 3). There was no apparent heterogeneity in our chosen SNPs, as evidenced by Cochran’s *Q* test (Supplementary Table 11). We found a negative association of BW to CAD from the inverse-variance weighted (IVW) estimates (odds ratio [OR] = 0.68, 95% CI: 0.59 to 0.80, *p* = 1.57× 10^-6^), which was consistent with all other MR methods (Table 3 and Figure 3). MR leave-one-out sensitivity analysis demonstrated that there was no influence of outlying and/or pleiotropic (Supplementary Figure 4). However, in the opposite direction, we found no causal relationship from CAD to BW (Supplementary Table 12).

**Table 3:** Causal relationship from BW to CAD by Mendelian randomization analysis.

**Method**	**nSNP**	**OR (95%CI)**	***P_value***
Simple median	52	0.72 (0.61, 0.84)	2.89E-05
Weighted median	52	0.68 (0.62, 0.76)	1.33E-13
Weighted mode	52	0.70 (0.55, 0.89)	6.34E-03
Maximum likelihood	52	0.69 (0.62, 0.76)	8.57E-13
MR Egger	52	0.63 (0.39, 1.01)	6.22E-02
Inverse variance weighted	52	0.68 (0.59, 0.80)	1.57E-06

Abbreviations: nSNP, number of SNPs applied in the test; OR, odds ratio; 95%CI, 95% confidence interval. Detailed SNPs information are exhibited in Supplementary Table 10.

### Functional enrichment and protein-protein interaction analyses

We discovered significant enrichment of biological processes including “regulation of phospholipid metabolic process” (*p* = 1.10×10^-4^) and “negative regulation of lipid transport” (*p* = 2.40×10^-4^) for genes associated with CAD by conducting functional enrichment analysis. Moreover, genes associated with BW were enriched in gene ontology (GO) terms like “tube morphogenesis” (*p* = 1.20×10^-4^) and “regulation of multicellular organismal process” (*p* = 3.10×10^-4^). Interestingly, the results for pleiotropic variants showed a cluster of biological processes in insulin and kinase categories, which might contribute to body growth and the progression of CAD (Table 4).

**Table 4:** Gene ontology (GO) terms enriched for SNP-annotated genes with FDR ≤ 0.05.

**Traits**	**GO terms**	**Term description**	**Gene counts**	**FDR**
CAD	GO:1903725	regulation of phospholipid metabolic process	7	1.10E-04
	GO:0032369	negative regulation of lipid transport	5	2.40E-04
	GO:0019220	regulation of phosphate metabolic process	22	2.70E-04
	GO:0032375	negative regulation of cholesterol transport	4	2.70E-04
	GO:0051241	negative regulation of multicellular organismal process	18	2.70E-04
BW	GO:0035239	tube morphogenesis	19	1.20E-04
	GO:0051239	regulation of multicellular organismal process	42	3.10E-04
	GO:0030154	cell differentiation	47	4.30E-04
	GO:0035295	tube development	20	4.30E-04
	GO:0072359	circulatory system development	20	4.30E-04
Pleiotropic	GO:0043560	insulin receptor substrate binding	3	1.20E-04
	GO:0005158	insulin receptor binding	3	4.30E-04
	GO:0043559	insulin binding	2	2.20E-03
	GO:0016538	cyclin-dependent protein serine/threonine kinase regulator activity	2	3.60E-02
	GO:0043548	phosphatidylinositol 3-kinase binding	2	3.60E-02

According to the protein-protein interaction network for CAD (Supplementary Figure 5A), proteins such as FURIN, FLT1, PLG, LDLR, and APOE were closely connected, and have been demonstrated to affect cardiovascular function [14, 40–42]. Similarly, in the BW network (Supplementary Figure 5B), proteins including ADRB1, ADCY5, ESR1, EPAS1*,* and CDKAL1 were closely connected and have been demonstrated to affect BW [21, 43–45].

## DISCUSSION

In this study, we incorporated summary statistics from two independent GWAS datasets and discovered 109 and 203 SNPs associated with CAD and BW, respectively. By performing the ccFDR method, we further detected 26 pleiotropic loci associated with both phenotypes. Following a bi-directional MR analysis and functional annotation, we confirmed the causal relationship from BW to CAD and speculated the underlying shared genetic mechanisms between these two traits.

Notably, we identified 11 CAD- and/or BW-associated SNPs that showed more than three of the metaQTL, pQTL, meQTL, or eQTL effects. These functional loci might have a great effect on the pathogenesis of CAD and/or BW. For example, rs11172113 is located in the intron of *LRP1*, a member of the low-density lipoprotein receptor family, which regulates extracellular proteolytic activities [46]. *LRP1* plays a pivotal role in mediating inflammation and efferocytosis [47], and mouse studies have shown that *LRP1* knockout leads to diminished vessel integrity and high-density lipoprotein secretion [48]. Another study proved that *LRP1* regulates food intake and energy homeostasis by acting as a co-activator of PPARγ [49]. Moreover, the lipidomic analysis demonstrated that the metabolite C18:1 sphingomyelin, which is associated with rs11172113, was enhanced in CAD patients compared to that in the control group [50]. Another longitudinal prospective study revealed that the alteration of sphingomyelin metabolism is associated with BW percentiles [51], suggesting a potentially crucial role for this SNP in both traits.

Furthermore, we identified one pleiotropic locus, rs10774625, showing metaQTL, pQTL, eQTL, and meQTL effects simultaneously. rs10774625 is located in the intron of *ATXN2*. One population-based GWAS demonstrated that the *ATXN2-SH3* region contributes to changes in the retinal venular caliber, an endophenotype of the microcirculation related to clinical cardiovascular diseases [52]. Animal experiments supported the role of *ATXN2* in translational regulation as well as embryonic development [53]. Another *ATXN2* knockdown experiment demonstrated that mice lacking *ATXN2* develop dysfunction in energy metabolism and weight regulation [54, 55]. It has been reported that rs10774625 is associated with the kynurenine metabolite pathway (KP) [56]. Evidence indicates that the activation of indoleamine 2,3-dioxygenase, the inducible enzyme in KP, is closely limited by endothelial cells [57], vascular smooth muscle cells [58], and dendritic cells [59], all of which play vital roles in cardiac pathophysiology [60]. Epidemiologically, it was shown that the concentration of kynurenine is associated with body weight indexes in a European cohort of more than 1000 people [61]. An immunohistochemistry study also detected that the kynurenine-to-tryptophan ratio limits the expression of inflammatory markers in the adipose tissue, which is correlated with body weight [62]. In addition, beta-2-microglobulin (B2M) is associated with rs10774625, which reduces the capacity for energy conversion and restricts intrauterine growth, resulting in low BW [63], and is also implicated in the pathogenesis of CAD [64]. These facts indicated that rs10774625 (representing gene *ATXN2*) might be essential in linking the pathogenesis between CAD and BW.

According to the functional enrichment results, we could also hypothesize the possible shared pathogenesis mechanisms between CAD and BW. GO terms including “regulation of phospholipid metabolic process”, “regulation of multicellular organismal process”, and “insulin receptor binding,” have important impacts on metabolic abnormalities, such as impaired fasting glucose [65], dyslipidemia, and hypertension [66], which could contribute to the increased risk for both traits.

Our study has some strengths. First, we improved the identification of potential CAD- and BW-associated SNPs and detected several pleiotropic loci in both traits. Following MR analysis, we assessed the causal effect between these two related traits. Second, we took into account ASD, which is unlikely to be correlated with CAD and BW, for a “control trait” enrichment analysis, which provided a baseline to examine pleiotropic enrichment and statistically validate the novel findings in our study. Third, evidence from metaQTL, pQTL, eQTL, and meQTL effects suggested a possible explanation for the etiology of CAD and/or BW and improved the interpretability of the results.

Additionally, our study includes some limitations. First, we were unable to link the genetic findings to clinical measures due to the lack of raw datasets for individual clinical outcomes. However, our study aimed to identify potential novel SNPs and explore the overlapping biological mechanisms between CAD and BW. We hope that our findings can be validated via functional experiments or fine-mapping studies. Second, although we confirmed the causal relationship from BW to CAD, the causalities of metabolomics, proteomics, and methylation between these two traits are unclear. Nevertheless, this problem could be solved by a follow-up multivariable MR study.

## CONCLUSIONS

In conclusion, by applying the cFDR and bi-directional MR analyses to two strongly associated traits, we detected significant pleiotropic SNPs of potential functions for CAD and/or BW and estimated the causal relationship from BW to CAD. These findings provide a better understanding of the shared genetic mechanisms between CAD and BW, which might suggest a novel research direction for early disease prevention and subsequent treatment.

## MATERIALS AND METHODS

### GWAS data sources

The first CAD GWAS was obtained from the Coronary Artery Disease Genome-wide Replication and Meta-analysis plus The Coronary Artery Disease Genetics (CARDIoGRAMplusC4D) Consortium. This meta-analysis of 48 multiple ancestry studies involved more than 8.6 million SNPs from 60,801 cases and 123,504 controls [18]. The first BW dataset conducted by the Early Growth Genetics (EGG) Consortium consisted of 45 multiple ancestry studies including 321,223 subjects. As the control trait, the ASD dataset, collected by the Psychiatric Genomics Consortium, contained 15,954 participants with European ancestry (7,387 ASD cases and 8,567 controls) [67]. For validation, two other CAD and BW datasets were used. The validation CAD dataset, comprising 10,801 cases and 137,914 controls, was collected by the CARDIoGRAMplusC4D Consortium [17]. The validation BW dataset, including 153,781 subjects, was collected by the EGG Consortium [21]. All datasets contained the summary statistics of each locus and the conducted genomic control [17, 18, 21, 22, 67].

### cFDR and ccFDR for identifying shared variants

### Data processing

First, two GWAS datasets were combined and 8,285,296 common SNPs with summary statistics remained for both CAD and BW phenotypes. Then, we performed LD-based pruning (r^2^ ≤ 0.2) using HapMap III genotypes as a reference, and the SNP of the pair with longer allele frequency was retained [31, 68]. After merging and pruning, 141,779 variants were prepared for further analysis.

### Pleiotropic enrichment evaluation

We constructed stratified Q-Q plots to estimate the pleiotropic enrichment in two related phenotypes using the “ggplot2” R package. In this study, -log_10_(*p*) which means the nominal *p*-value and -log_10_(*q*) which means the empirical quantile were plotted on the Y- and X-axes, respectively, at different significance levels (*p* ≤ 1, *p* ≤ 0.1, *p* ≤ 0.01, *p* ≤ 0.001, and *p* ≤ 0.0001). Under the null hypothesis, plots would fall on the line Y=X, and the enrichment of pleiotropic loci could be evaluated by the degree of leftward deviation from the null line. Additionally, we constructed fold-enrichment plots as a supplement for the Q-Q plots. Fold-enrichment and -log_10_(*p*) were plotted on the Y- and X-axes, respectively, at different significance levels (*p* ≤ 1, *p* ≤ 0.1, *p* ≤ 0.01, and *p* ≤ 0.001) for CAD and BW. Pleiotropy could be visually observed via an upward deflection from the baseline (for the group including all SNPs (*p* = 1)).

### Calculation of cFDR and ccFDR values

The cFDR method was used to estimate the possibility that a random SNP was not associated with the primary trait, given that its strength for the conditional traits was below the threshold [28]. This was an extension of the original FDR framework, applied for the cross-trait analysis [69]. Specifically, we computed cFDR for each SNP, selecting CAD as the primary phenotype given its association with BW (CAD|BW) and vice versa (BW|CAD). To detect the pleiotropic loci for both traits, we calculated the ccFDR value, the maximum of the two cFDR values. The ccFDR value indicated that the possibility that a given SNP was false positively related to two traits (CAD and BW) simultaneously. The thresholds for cFDR and ccFDR were set at 0.05. Detailed steps of this approach have been described by Andreassen et al. [29].

### Bi-directional MR analysis

To determine the relationship between BW and CAD, we performed a bi-directional MR analysis using the “TwoSampleMR” R package [70]. First, SNPs that were genome-wide significant (*p* ≤ 5x10^-8^) in the exposure GWAS dataset were selected as genetic variants. To ensure that the instruments for exposure were independent, we performed LD-based clumping (r^2^ > 0.001) and only retained the SNP with a lower *p*-value [68, 71]. Then, we extracted summary-level statistics for each selected SNP from the outcome trait and removed the SNPs related to the outcome phenotype (*p* ≤ 5x10^-8^). The summary associations of candidate genetic variants were harmonized as described previously [72]. Finally, MR was conducted using IVW, simple median, weighted median, weighted mode, maximum likelihood, and MR-Egger approaches. BW and CAD were used as exposure and outcome measures, respectively, to identify the causal direction. The datasets used in the MR analysis were the same as that in the original cFDR analysis (The first CAD and BW datasets). To investigate whether any SNP had an outlying and/or pleiotropic influence, we also performed a leave-one-out sensitivity analysis.

### Functional annotation and protein-protein interaction analyses

Online tools HaploReg (http://compbio.mit.edu/HaploReg) and RegulomeDB (http://www.regulomedb.org/) were applied to map each of the identified significant SNPs to nearby genes, corresponding DNA features, and regulatory elements. Next, we detected whether they possessed metaQTL, pQTL, meQTL, or eQTL effects. To obtain the metaQTL and pQTL hits, we applied the web-based software SNiPA (http://www.snipa.org/), meQTL and eQTL information were collected from Bonder’s study [73] and HaploReg, respectively.

We used the GOEAST software to detect statistically overrepresented GO terms within the selected gene sets [74]. Meanwhile, using the STRING database, we conducted protein-protein interaction analyses to investigate the interaction and functional relationships of the identified CAD- and/or BW-related genes [75].

## Supplementary Material

Supplementary Figures

Supplementary Table 1

Supplementary Table 2

Supplementary Table 3

Supplementary Table 4

Supplementary Table 5

Supplementary Table 6

Supplementary Tables 7, 8, 9, 10, 11 and 12
