# Supplementary material for: Identification of novel SNPs associated with coronary artery disease and birth weight using a pleiotropic cFDR method
Source: Aging (Albany NY). 2020 Dec 19;13(3):3618–44. doi: 10.18632/aging.202322 (PMC7906162; doi:10.18632/aging.202322)
Supplement: Supplementary Figures [file aging-13-202322-s001.pdf]

SUPPLEMENTARY FIGURES

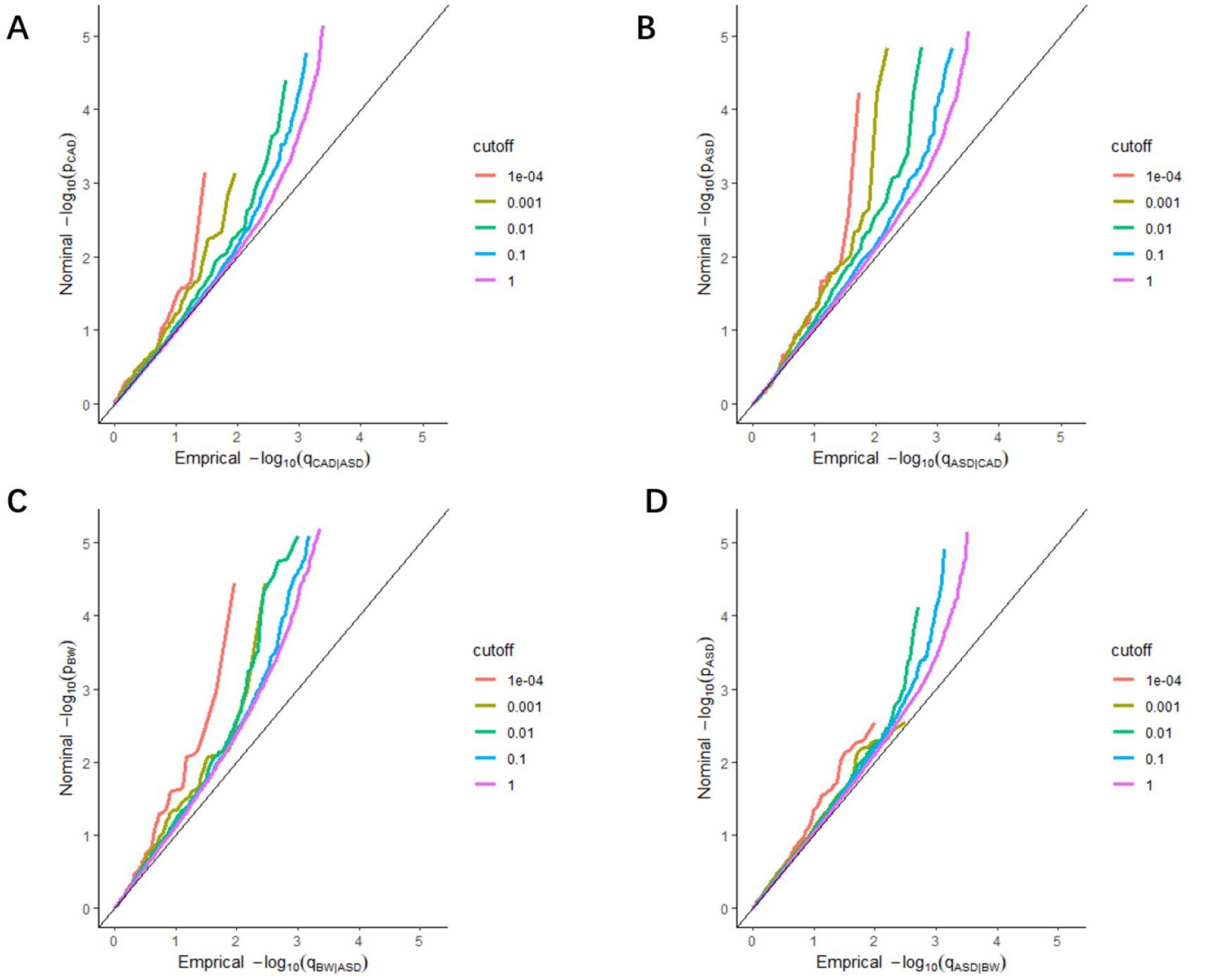

Supplementary Figure 1. Stratified Q-Q plots. Stratified Q-Q plots of nominal vs. empirical -log10(*p*) values in principal trait below the standard GWAS threshold of $p \leq 5 \times 10^{-8}$ as a function of the significance of the association with conditional trait at the level of $p \leq 1$, $p \leq 0.1$, $p \leq 0.01$, $p \leq 0.001$, and $p \leq 0.0001$, respectively. (**A**) CAD as a function of the significance of the association with ASD, (**B**) ASD as a function of the significance of the association with CAD, (**C**) BW as a function of the significance of the association with ASD, and (**D**) ASD as a function of the significance of the association with BW.

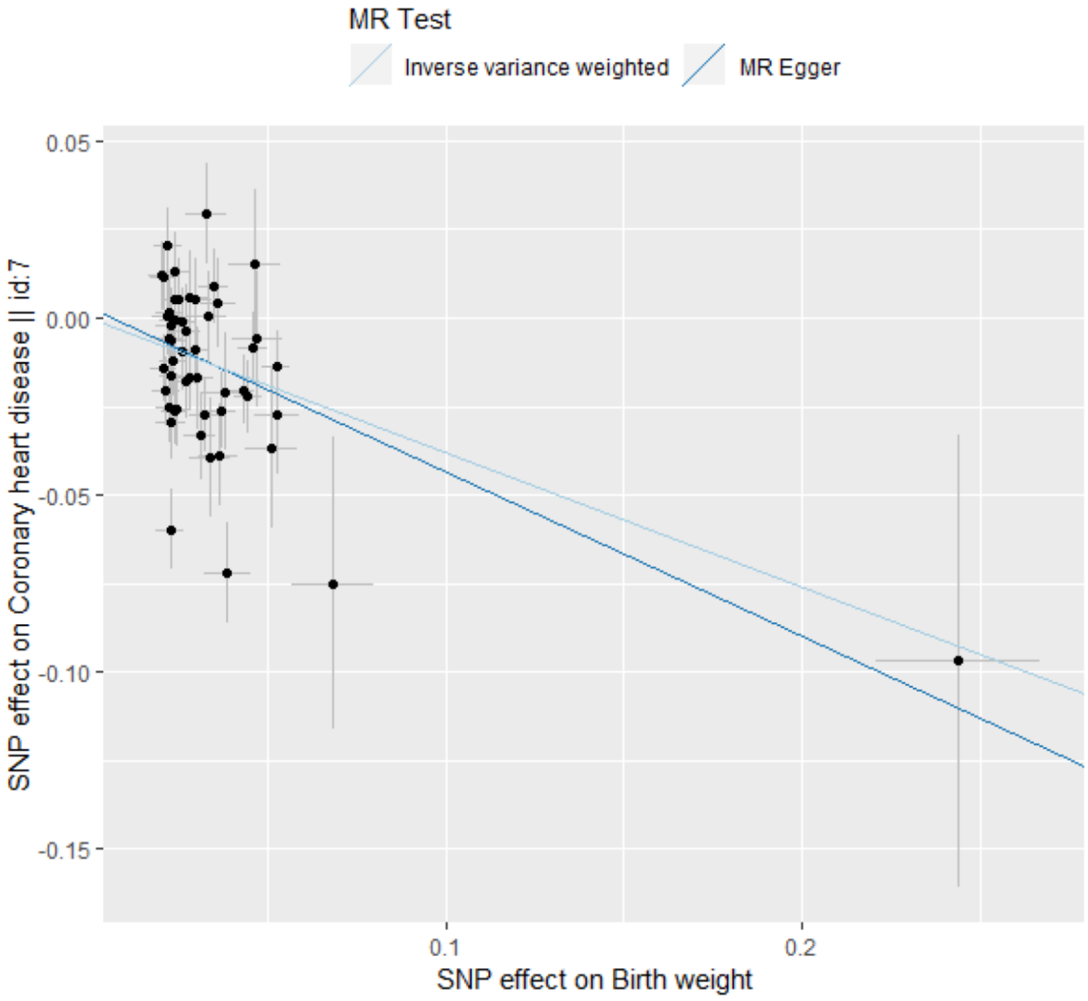

Supplementary Figure 2. MR regression scatter plot. The relationship of the SNP effects on the exposure against the SNP effects on the outcome was depicted using a scatter plot. The different color lines show the results of corresponding MR analysis methods.

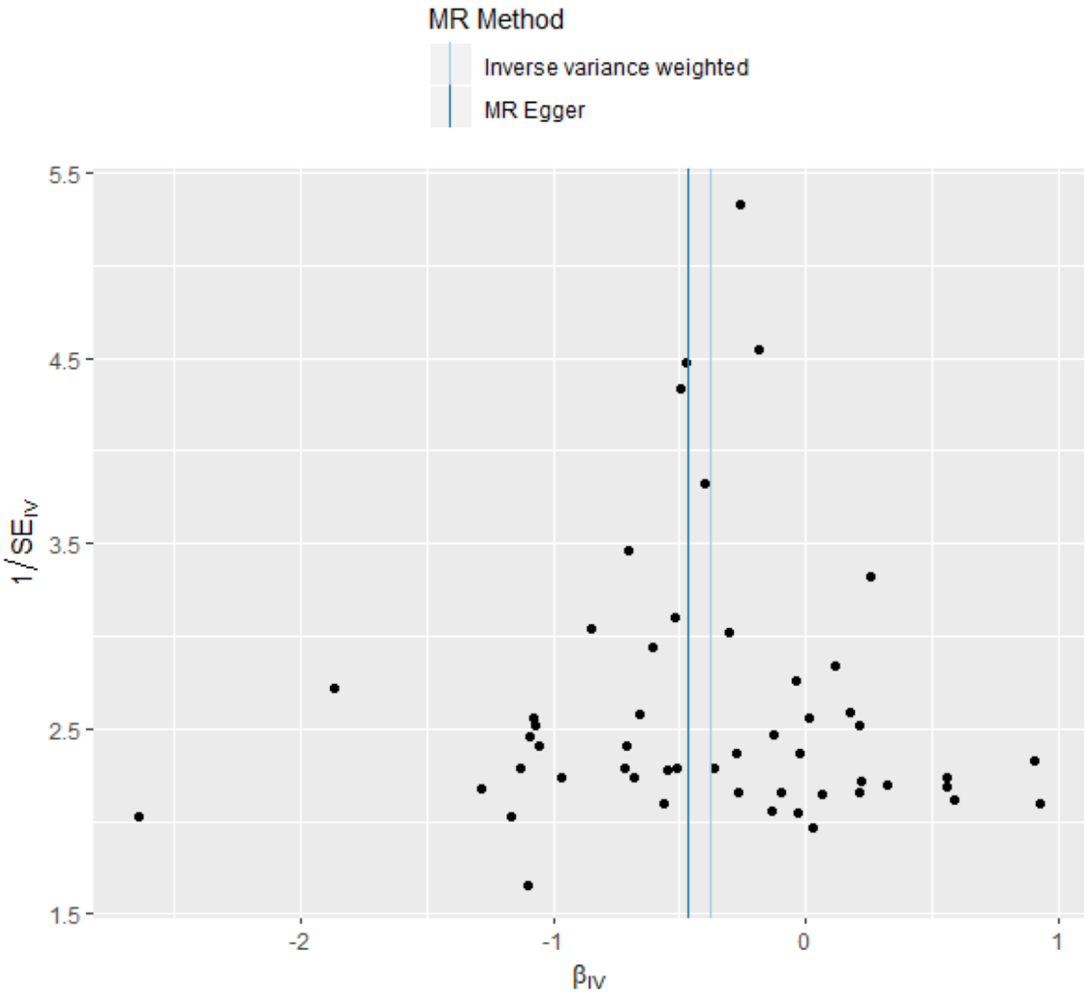

Supplementary Figure 3. MR regression funnel plot. Asymmetry in a funnel plot is useful for gauging the reliability of a particular MR analysis Each SNP's MR estimate is plotted against its minor allele frequency (MAF) corrected association with BW. A MAF correction proportional to the BW related SNP standard error is used since a low-frequency allele is likely to be measured with low precision. Similar to the use of funnel plots in the meta-analysis literature, this plot can be used for visual inspection of symmetry, where any deviation can be suggestive of pleiotropy.

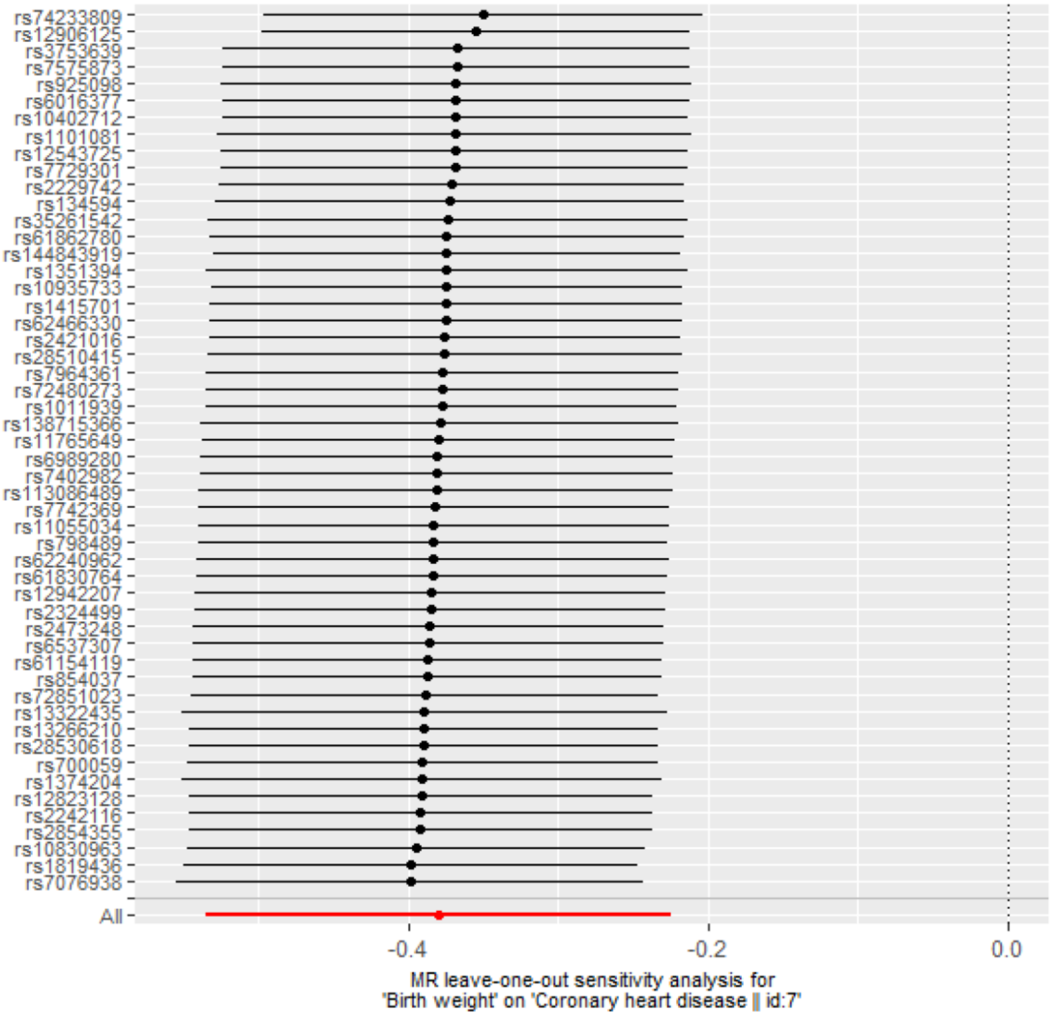

Supplementary Figure 4. Leave-one-out plot. The leave-one-out analysis result was visualized by the forest plot. Each black dot and line correspond to the effect size and 95% confidence interval (95%CI), and the bottom red dot and line is the overall effect size and 95%CI.

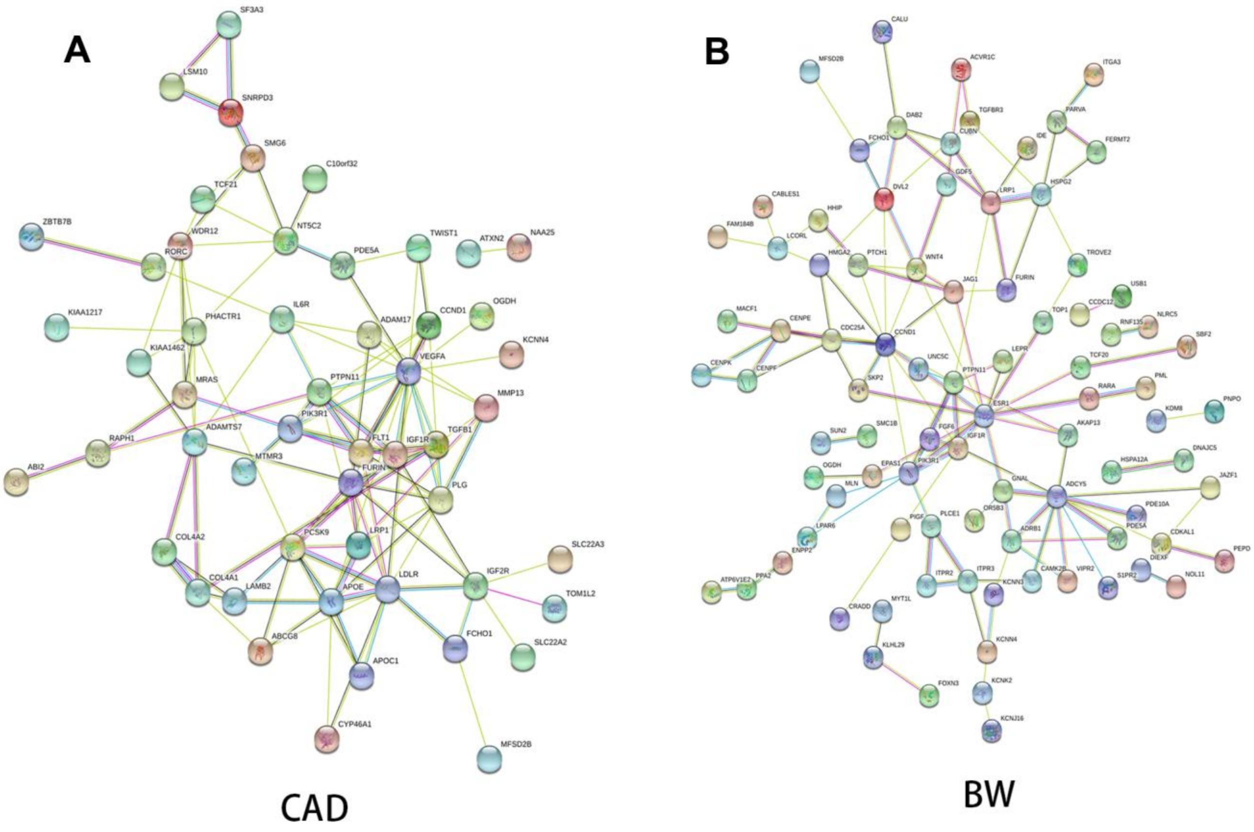

Supplementary Figure 5. Functional protein association network analysis. Connections are based on co-expression and experimental evidence with a STRING 10.5 summary score above 0.4. Each filled node denotes a gene; edges between nodes indicate protein-protein interactions between protein products of the corresponding genes in (**A**) CAD and (**B**) BW. Different edge colors represent the types of evidence for the association.
