# Supplementary material for: Identification of novel SNPs associated with coronary artery disease and birth weight using a pleiotropic cFDR method
Source: Aging (Albany NY). 2020 Dec 19;13(3):3618–44. doi: 10.18632/aging.202322 (PMC7906162; doi:10.18632/aging.202322)
Supplement: Supplementary Table 1 [file aging-13-202322-s002.docx]

**Supplementary Table 1.** Conditional FDR values of 109 SNPs for CAD given the BW (cFDR ≤ 0.05).

| **SNP** | **Chr** | **Pos** | **Alt** | **Gene** | **Annotation** | **SNP Type** | **Gene Type** | ***P*_value** | **cFDR** |
| --- | --- | --- | --- | --- | --- | --- | --- | --- | --- |
| [rs10080815](https://pubs.broadinstitute.org/mammals/haploreg/detail_v4.1.php?query=&id=rs10080815) | 6 | 160266380 | G/C | *SLC22A2* | intergenic | Novel | Novel | 2.78E-10 | 2.98E-06 |
| [rs1029212](https://pubs.broadinstitute.org/mammals/haploreg/detail_v4.1.php?query=&id=rs1029212) | 6 | 133850341 | G/C | *RP4-662A9.2* | intronic | Confirmed | Novel | 1.18E-03 | 4.40E-02 |
| [rs1047418](https://pubs.broadinstitute.org/mammals/haploreg/detail_v4.1.php?query=&id=rs1047418) | 14 | 75139089 | G/C | *TMED10* | intronic | Novel | Novel | 1.04E-17 | 3.94E-13 |
| [rs10774625](https://pubs.broadinstitute.org/mammals/haploreg/detail_v4.1.php?query=&id=rs10774625) | 12 | 111472415 | G/C | *ATXN2* | intronic | Confirmed | Confirmed | 3.35E-05 | 3.75E-02 |
| [rs10781976](https://pubs.broadinstitute.org/mammals/haploreg/detail_v4.1.php?query=&id=rs10781976) | 16 | 75280940 | T/A | *U6* | intergenic | Novel | Novel | 6.35E-05 | 3.85E-02 |
| [rs10791643](https://pubs.broadinstitute.org/mammals/haploreg/detail_v4.1.php?query=&id=rs10791643) | 11 | 103803611 | G/C | *RP11-563P16.1* | intergenic | Novel | Novel | 1.43E-06 | 4.84E-03 |
| [rs10818580](https://pubs.broadinstitute.org/mammals/haploreg/detail_v4.1.php?query=&id=rs10818580) | 9 | 121652743 | A/T | *DAB2IP* | intronic | Novel | Novel | 6.19E-07 | 1.70E-03 |
| [rs10965212](https://pubs.broadinstitute.org/mammals/haploreg/detail_v4.1.php?query=&id=rs10965212) | 9 | 22023796 | A/T | *RP11-145E5.5* | intronic | Confirmed | Novel | 2.78E-04 | 3.95E-02 |
| [rs10965228](https://pubs.broadinstitute.org/mammals/haploreg/detail_v4.1.php?query=&id=rs10965228) | 9 | 22082381 | G/C | *CDKN2B-AS1* | intronic | Novel | Novel | 1.18E-04 | 1.37E-03 |
| [rs11066301](https://pubs.broadinstitute.org/mammals/haploreg/detail_v4.1.php?query=&id=rs11066301) | 12 | 112433568 | G/C | *PTPN11* | intronic | Confirmed | Confirmed | 3.58E-07 | 1.87E-03 |
| [rs11079045](https://pubs.broadinstitute.org/mammals/haploreg/detail_v4.1.php?query=&id=rs11079045) | 17 | 42435651 | A/T | *U7* | intergenic | Novel | Novel | 2.84E-05 | 4.89E-02 |
| [rs11172113](https://pubs.broadinstitute.org/mammals/haploreg/detail_v4.1.php?query=&id=rs11172113) | 12 | 57133500 | C/G | *LRP1* | intronic | Novel | Novel | 1.49E-04 | 5.73E-03 |
| [rs11206803](https://pubs.broadinstitute.org/mammals/haploreg/detail_v4.1.php?query=&id=rs11206803) | 1 | 56411837 | T/A | *RP4-710M16.2* | intergenic | Novel | Novel | 4.63E-05 | 4.50E-02 |
| [rs11238956](https://pubs.broadinstitute.org/mammals/haploreg/detail_v4.1.php?query=&id=rs11238956) | 10 | 44254406 | C/G | *RP11-20J15.2* | intergenic | Novel | Novel | 2.46E-05 | 2.05E-02 |
| [rs11244035](https://pubs.broadinstitute.org/mammals/haploreg/detail_v4.1.php?query=&id=rs11244035) | 9 | 133205932 | T/A | *OBP2B* | missense | Novel | Novel | 1.88E-06 | 5.48E-03 |
| [rs11591147](https://pubs.broadinstitute.org/mammals/haploreg/detail_v4.1.php?query=&id=rs11591147) | 1 | 55039974 | T/A | *PCSK9* | missense | Novel | Confirmed | 6.87E-05 | 4.95E-02 |
| [rs11601507](https://pubs.broadinstitute.org/mammals/haploreg/detail_v4.1.php?query=&id=rs11601507) | 11 | 5679844 | A/T | *TRIM5* | missense | Novel | Novel | 5.28E-05 | 8.98E-04 |
| [rs11617955](https://pubs.broadinstitute.org/mammals/haploreg/detail_v4.1.php?query=&id=rs11617955) | 13 | 110165755 | A/T | *COL4A1* | intronic | Novel | Confirmed | 2.21E-05 | 4.18E-02 |
| [rs11668477](https://pubs.broadinstitute.org/mammals/haploreg/detail_v4.1.php?query=&id=rs11668477) | 19 | 11084354 | G/C | *LDLR* | intergenic | Novel | Confirmed | 3.34E-07 | 1.69E-03 |
| [rs11675251](https://pubs.broadinstitute.org/mammals/haploreg/detail_v4.1.php?query=&id=rs11675251) | 2 | 203384676 | G/C | *ABI2* | intronic | Novel | Novel | 5.71E-07 | 4.07E-04 |
| [rs12044531](https://pubs.broadinstitute.org/mammals/haploreg/detail_v4.1.php?query=&id=rs12044531) | 1 | 37987369 | A/T | *SF3A3* | intronic | Novel | Novel | 5.46E-06 | 1.39E-02 |
| [rs12148530](https://pubs.broadinstitute.org/mammals/haploreg/detail_v4.1.php?query=&id=rs12148530) | 15 | 96542056 | C/G | *7SK* | intergenic | Novel | Novel | 1.80E-11 | 3.79E-07 |
| [rs12306172](https://pubs.broadinstitute.org/mammals/haploreg/detail_v4.1.php?query=&id=rs12306172) | 12 | 54145221 | A/T | *RP11-834C11.11* | intergenic | Novel | Novel | 4.12E-05 | 4.76E-02 |
| [rs12474540](https://pubs.broadinstitute.org/mammals/haploreg/detail_v4.1.php?query=&id=rs12474540) | 2 | 9559184 | C/G | *ADAM17* | intergenic | Novel | Novel | 2.83E-05 | 4.95E-02 |
| [rs12530920](https://pubs.broadinstitute.org/mammals/haploreg/detail_v4.1.php?query=&id=rs12530920) | 7 | 19016174 | C/G | *TWIST1* | intergenic | Novel | Novel | 5.86E-07 | 2.48E-03 |
| [rs12792912](https://pubs.broadinstitute.org/mammals/haploreg/detail_v4.1.php?query=&id=rs12792912) | 11 | 102930574 | G/C | *MMP13* | intergenic | Novel | Novel | 2.69E-10 | 1.48E-08 |
| [rs12943500](https://pubs.broadinstitute.org/mammals/haploreg/detail_v4.1.php?query=&id=rs12943500) | 17 | 17887274 | T/A | *TOM1L2* | intronic | Confirmed | Confirmed | 9.20E-09 | 7.37E-05 |
| [rs13035774](https://pubs.broadinstitute.org/mammals/haploreg/detail_v4.1.php?query=&id=rs13035774) | 2 | 24135782 | T/A | *AC008073.6* | intronic | Novel | Novel | 9.77E-06 | 1.49E-02 |
| [rs13070927](https://pubs.broadinstitute.org/mammals/haploreg/detail_v4.1.php?query=&id=rs13070927) | 3 | 14878139 | T/A | *FGD5* | intronic | Novel | Novel | 5.12E-06 | 8.58E-03 |
| [rs1319869](https://pubs.broadinstitute.org/mammals/haploreg/detail_v4.1.php?query=&id=rs1319869) | 15 | 98669256 | T/A | *IGF1R* | intronic | Novel | Novel | 1.71E-05 | 3.56E-03 |
| [rs1333050](https://pubs.broadinstitute.org/mammals/haploreg/detail_v4.1.php?query=&id=rs1333050) | 9 | 22125914 | T/A | *CDKN2B-AS1* | intergenic | Confirmed | Novel | 2.69E-14 | 6.41E-10 |
| [rs13382133](https://pubs.broadinstitute.org/mammals/haploreg/detail_v4.1.php?query=&id=rs13382133) | 19 | 17748127 | T/A | *FCHO1* | intronic | Novel | Novel | 4.50E-06 | 3.43E-03 |
| [rs1418278](https://pubs.broadinstitute.org/mammals/haploreg/detail_v4.1.php?query=&id=rs1418278) | 10 | 30009149 | G/C | *KIAA1462* | intergenic | Novel | Confirmed | 3.41E-06 | 6.43E-03 |
| [rs1433099](https://pubs.broadinstitute.org/mammals/haploreg/detail_v4.1.php?query=&id=rs1433099) | 19 | 11131982 | C/G | *LDLR* | 3'-UTR | Novel | Confirmed | 7.41E-06 | 1.63E-02 |
| [rs1480933](https://pubs.broadinstitute.org/mammals/haploreg/detail_v4.1.php?query=&id=rs1480933) | 4 | 119512093 | T/A | *PDE5A* | intronic | Novel | Novel | 1.00E-06 | 1.55E-03 |
| [rs1482472](https://pubs.broadinstitute.org/mammals/haploreg/detail_v4.1.php?query=&id=rs1482472) | 10 | 44187525 | C/G | *RP11-20J15.2* | intergenic | Novel | Novel | 3.40E-05 | 4.91E-02 |
| [rs1541853](https://pubs.broadinstitute.org/mammals/haploreg/detail_v4.1.php?query=&id=rs1541853) | 2 | 202970454 | C/G | *WDR12* | intronic | Novel | Confirmed | 9.17E-08 | 5.79E-04 |
| [rs1547705](https://pubs.broadinstitute.org/mammals/haploreg/detail_v4.1.php?query=&id=rs1547705) | 9 | 22082376 | C/G | *CDKN2B-AS1* | intronic | Novel | Novel | 6.65E-08 | 4.97E-04 |
| [rs16891156](https://pubs.broadinstitute.org/mammals/haploreg/detail_v4.1.php?query=&id=rs16891156) | 6 | 160187772 | C/G | *SLC22A2* | intergenic | Novel | Novel | 3.55E-08 | 2.73E-04 |
| [rs16986953](https://pubs.broadinstitute.org/mammals/haploreg/detail_v4.1.php?query=&id=rs16986953) | 2 | 19742712 | A/T | *AC019055.1* | intergenic | Confirmed | Novel | 4.23E-04 | 2.64E-02 |
| [rs17477113](https://pubs.broadinstitute.org/mammals/haploreg/detail_v4.1.php?query=&id=rs17477113) | 2 | 203501730 | G/C | *RAPH1* | intronic | Novel | Novel | 5.94E-06 | 9.98E-03 |
| [rs1861044](https://pubs.broadinstitute.org/mammals/haploreg/detail_v4.1.php?query=&id=rs1861044) | 4 | 15537875 | G/C | *CC2D2A* | intronic | Novel | Novel | 6.82E-09 | 6.03E-05 |
| [rs1888657](https://pubs.broadinstitute.org/mammals/haploreg/detail_v4.1.php?query=&id=rs1888657) | 10 | 24544685 | C/G | *KIAA1217* | intronic | Novel | Novel | 1.74E-07 | 8.16E-04 |
| [rs2001945](https://pubs.broadinstitute.org/mammals/haploreg/detail_v4.1.php?query=&id=rs2001945) | 8 | 125465736 | C/G | *RP11-136O12.2* | intergenic | Novel | Novel | 2.28E-06 | 6.36E-03 |
| [rs2126202](https://pubs.broadinstitute.org/mammals/haploreg/detail_v4.1.php?query=&id=rs2126202) | 17 | 2192418 | A/T | *SMG6* | intronic | Novel | Confirmed | 1.45E-08 | 1.16E-04 |
| [rs2144723](https://pubs.broadinstitute.org/mammals/haploreg/detail_v4.1.php?query=&id=rs2144723) | 6 | 160700358 | T/A | *RP1-81D8.3* | intergenic | Novel | Novel | 2.78E-06 | 8.16E-03 |
| [rs2146238](https://pubs.broadinstitute.org/mammals/haploreg/detail_v4.1.php?query=&id=rs2146238) | 14 | 99706392 | G/C | *CYP46A1* | intronic | Confirmed | Confirmed | 3.70E-06 | 1.08E-02 |
| [rs2162042](https://pubs.broadinstitute.org/mammals/haploreg/detail_v4.1.php?query=&id=rs2162042) | 12 | 89725520 | A/T | *RN5S365* | intergenic | Novel | Novel | 1.09E-04 | 1.34E-03 |
| [rs2166529](https://pubs.broadinstitute.org/mammals/haploreg/detail_v4.1.php?query=&id=rs2166529) | 2 | 85515052 | T/A | *Metazoa_SRP* | intergenic | Novel | Novel | 2.22E-05 | 3.78E-02 |
| [rs2238151](https://pubs.broadinstitute.org/mammals/haploreg/detail_v4.1.php?query=&id=rs2238151) | 12 | 111774029 | C/G | *ALDH2* | intronic | Novel | Confirmed | 9.40E-08 | 5.98E-04 |
| [rs2268310](https://pubs.broadinstitute.org/mammals/haploreg/detail_v4.1.php?query=&id=rs2268310) | 7 | 44637499 | T/A | *OGDH* | intronic | Novel | Novel | 9.26E-04 | 4.76E-02 |
| [rs2298389](https://pubs.broadinstitute.org/mammals/haploreg/detail_v4.1.php?query=&id=rs2298389) | 22 | 24557381 | T/A | *SNRPD3* | intronic | Novel | Novel | 7.00E-06 | 1.58E-02 |
| [rs2306374](https://pubs.broadinstitute.org/mammals/haploreg/detail_v4.1.php?query=&id=rs2306374) | 3 | 138401110 | C/G | *MRAS* | intronic | Novel | Confirmed | 7.93E-05 | 1.41E-03 |
| [rs2327430](https://pubs.broadinstitute.org/mammals/haploreg/detail_v4.1.php?query=&id=rs2327430) | 6 | 133888899 | T/A | *TCF21* | intergenic | Novel | Confirmed | 4.00E-10 | 4.39E-06 |
| [rs2339940](https://pubs.broadinstitute.org/mammals/haploreg/detail_v4.1.php?query=&id=rs2339940) | 2 | 24028917 | T/A | *MFSD2B* | intergenic | Novel | Novel | 2.06E-07 | 3.45E-05 |
| [rs2802490](https://pubs.broadinstitute.org/mammals/haploreg/detail_v4.1.php?query=&id=rs2802490) | 10 | 44103565 | A/T | *AL512640.1* | intergenic | Novel | Novel | 8.76E-04 | 1.08E-02 |
| [rs2812](https://pubs.broadinstitute.org/mammals/haploreg/detail_v4.1.php?query=&id=rs2812) | 17 | 64323758 | C/G | *TEX2* | 3'-UTR | Novel | Novel | 7.10E-05 | 6.65E-03 |
| [rs2836631](https://pubs.broadinstitute.org/mammals/haploreg/detail_v4.1.php?query=&id=rs2836631) | 21 | 38693982 | G/C | *ERG* | intergenic | Novel | Novel | 5.85E-03 | 4.98E-02 |
| [rs3172494](https://pubs.broadinstitute.org/mammals/haploreg/detail_v4.1.php?query=&id=rs3172494) | 3 | 48694054 | T/A | *IP6K2* | 3'-UTR | Novel | Novel | 9.71E-06 | 2.04E-02 |
| [rs34759087](https://pubs.broadinstitute.org/mammals/haploreg/detail_v4.1.php?query=&id=rs34759087) | 3 | 49124851 | T/A | *LAMB2* | missense | Novel | Novel | 1.19E-03 | 3.15E-02 |
| [rs3751395](https://pubs.broadinstitute.org/mammals/haploreg/detail_v4.1.php?query=&id=rs3751395) | 13 | 28384818 | A/T | *FLT1* | intronic | Novel | Confirmed | 1.44E-04 | 4.14E-02 |
| [rs3754211](https://pubs.broadinstitute.org/mammals/haploreg/detail_v4.1.php?query=&id=rs3754211) | 1 | 150979381 | A/T | *ANXA9* | intergenic | Novel | Novel | 5.68E-06 | 9.86E-03 |
| [rs3756668](https://pubs.broadinstitute.org/mammals/haploreg/detail_v4.1.php?query=&id=rs3756668) | 5 | 68300260 | A/T | *PIK3R1* | 3'-UTR | Novel | Novel | 7.08E-04 | 3.85E-02 |
| [rs3811417](https://pubs.broadinstitute.org/mammals/haploreg/detail_v4.1.php?query=&id=rs3811417) | 1 | 151832431 | C/G | *RORC* | intergenic | Novel | Novel | 4.29E-04 | 2.58E-02 |
| [rs3918291](https://pubs.broadinstitute.org/mammals/haploreg/detail_v4.1.php?query=&id=rs3918291) | 6 | 160407110 | C/G | *SLC22A3* | synonymous | Novel | Confirmed | 4.22E-06 | 1.02E-02 |
| [rs405509](https://pubs.broadinstitute.org/mammals/haploreg/detail_v4.1.php?query=&id=rs405509) | 19 | 44905579 | G/C | *APOE* | intergenic | Novel | Confirmed | 7.76E-32 | 3.40E-27 |
| [rs4233701](https://pubs.broadinstitute.org/mammals/haploreg/detail_v4.1.php?query=&id=rs4233701) | 2 | 23706216 | C/G | *KLHL29* | intronic | Novel | Novel | 3.00E-05 | 2.12E-02 |
| [rs4245791](https://pubs.broadinstitute.org/mammals/haploreg/detail_v4.1.php?query=&id=rs4245791) | 2 | 43847292 | T/A | *ABCG8* | intronic | Novel | Confirmed | 2.66E-06 | 1.86E-03 |
| [rs4290163](https://pubs.broadinstitute.org/mammals/haploreg/detail_v4.1.php?query=&id=rs4290163) | 10 | 102851169 | T/A | *C10orf32* | intergenic | Novel | Novel | 5.53E-04 | 5.26E-03 |
| [rs4420638](https://pubs.broadinstitute.org/mammals/haploreg/detail_v4.1.php?query=&id=rs4420638) | 19 | 44919689 | G/C | *APOC1* | intergenic | Novel | Confirmed | 1.45E-07 | 3.77E-06 |
| [rs445925](https://pubs.broadinstitute.org/mammals/haploreg/detail_v4.1.php?query=&id=rs445925) | 19 | 44912383 | A/T | *APOC1* | intergenic | Confirmed | Confirmed | 1.06E-05 | 1.00E-02 |
| [rs4481859](https://pubs.broadinstitute.org/mammals/haploreg/detail_v4.1.php?query=&id=rs4481859) | 1 | 222559314 | G/C | *TAF1A* | intronic | Novel | Novel | 8.12E-04 | 4.43E-02 |
| [rs4627080](https://pubs.broadinstitute.org/mammals/haploreg/detail_v4.1.php?query=&id=rs4627080) | 11 | 9314802 | G/C | *TMEM41B* | intergenic | Novel | Novel | 1.39E-05 | 2.80E-02 |
| [rs4643791](https://pubs.broadinstitute.org/mammals/haploreg/detail_v4.1.php?query=&id=rs4643791) | 4 | 119344464 | A/T | *FABP2* | intergenic | Novel | Novel | 4.51E-05 | 5.22E-03 |
| [rs4767293](https://pubs.broadinstitute.org/mammals/haploreg/detail_v4.1.php?query=&id=rs4767293) | 12 | 112025492 | G/C | *NAA25* | intergenic | Novel | Confirmed | 1.00E-06 | 3.71E-03 |
| [rs4773144](https://pubs.broadinstitute.org/mammals/haploreg/detail_v4.1.php?query=&id=rs4773144) | 13 | 110308365 | G/C | *COL4A2* | intronic | Confirmed | Confirmed | 2.31E-04 | 2.02E-02 |
| [rs4803455](https://pubs.broadinstitute.org/mammals/haploreg/detail_v4.1.php?query=&id=rs4803455) | 19 | 41345604 | A/T | *TGFB1* | intronic | Novel | Novel | 1.40E-07 | 2.42E-04 |
| [rs4845618](https://pubs.broadinstitute.org/mammals/haploreg/detail_v4.1.php?query=&id=rs4845618) | 1 | 154427539 | T/A | *IL6R* | intronic | Novel | Confirmed | 9.33E-10 | 1.09E-05 |
| [rs4895390](https://pubs.broadinstitute.org/mammals/haploreg/detail_v4.1.php?query=&id=rs4895390) | 6 | 133839976 | C/G | *RP4-662A9.2* | intronic | Novel | Novel | 3.71E-07 | 1.86E-03 |
| [rs502467](https://pubs.broadinstitute.org/mammals/haploreg/detail_v4.1.php?query=&id=rs502467) | 3 | 172009573 | C/G | *FNDC3B* | intergenic | Novel | Novel | 1.11E-03 | 1.32E-02 |
| [rs583489](https://pubs.broadinstitute.org/mammals/haploreg/detail_v4.1.php?query=&id=rs583489) | 10 | 44243240 | G/C | *RP11-20J15.2* | intergenic | Novel | Novel | 3.82E-07 | 1.54E-03 |
| [rs6006426](https://pubs.broadinstitute.org/mammals/haploreg/detail_v4.1.php?query=&id=rs6006426) | 22 | 30273894 | A/T | *MTMR3* | intergenic | Novel | Novel | 3.62E-05 | 4.12E-02 |
| [rs611003](https://pubs.broadinstitute.org/mammals/haploreg/detail_v4.1.php?query=&id=rs611003) | 11 | 69630516 | A/T | *CCND1* | intergenic | Novel | Novel | 1.43E-06 | 1.66E-03 |
| [rs624249](https://pubs.broadinstitute.org/mammals/haploreg/detail_v4.1.php?query=&id=rs624249) | 6 | 160258368 | A/T | *SLC22A2* | synonymous | Novel | Novel | 2.47E-11 | 3.56E-07 |
| [rs630014](https://pubs.broadinstitute.org/mammals/haploreg/detail_v4.1.php?query=&id=rs630014) | 9 | 133274306 | G/C | *ABO* | intronic | Novel | Confirmed | 1.88E-41 | 3.19E-37 |
| [rs655246](https://pubs.broadinstitute.org/mammals/haploreg/detail_v4.1.php?query=&id=rs655246) | 1 | 109289661 | G/C | *MYBPHL* | intergenic | Novel | Novel | 1.94E-05 | 3.32E-02 |
| [rs656461](https://pubs.broadinstitute.org/mammals/haploreg/detail_v4.1.php?query=&id=rs656461) | 6 | 149477699 | T/A | *ZC3H12D* | intronic | Novel | Novel | 7.07E-11 | 1.54E-06 |
| [rs6673081](https://pubs.broadinstitute.org/mammals/haploreg/detail_v4.1.php?query=&id=rs6673081) | 1 | 155017119 | C/G | *ZBTB7B* | 3'-UTR | Novel | Novel | 5.10E-07 | 1.87E-03 |
| [rs670950](https://pubs.broadinstitute.org/mammals/haploreg/detail_v4.1.php?query=&id=rs670950) | 19 | 43777410 | C/G | *KCNN4* | intronic | Novel | Novel | 9.18E-05 | 2.94E-02 |
| [rs6713510](https://pubs.broadinstitute.org/mammals/haploreg/detail_v4.1.php?query=&id=rs6713510) | 2 | 226169783 | A/T | *AC068138.1* | intronic | Confirmed | Novel | 3.02E-09 | 2.56E-05 |
| [rs688359](https://pubs.broadinstitute.org/mammals/haploreg/detail_v4.1.php?query=&id=rs688359) | 6 | 160044259 | A/T | *IGF2R* | intronic | Novel | Novel | 7.47E-06 | 1.82E-02 |
| [rs6922782](https://pubs.broadinstitute.org/mammals/haploreg/detail_v4.1.php?query=&id=rs6922782) | 6 | 12948156 | A/T | *PHACTR1* | intronic | Novel | Confirmed | 3.94E-06 | 9.48E-03 |
| [rs7092200](https://pubs.broadinstitute.org/mammals/haploreg/detail_v4.1.php?query=&id=rs7092200) | 10 | 103085115 | C/G | *NT5C2* | intergenic | Novel | Confirmed | 9.03E-06 | 9.14E-03 |
| [rs7164299](https://pubs.broadinstitute.org/mammals/haploreg/detail_v4.1.php?query=&id=rs7164299) | 15 | 89036690 | C/G | *RP11-326A19.4* | intergenic | Novel | Novel | 6.04E-06 | 1.48E-02 |
| [rs7168915](https://pubs.broadinstitute.org/mammals/haploreg/detail_v4.1.php?query=&id=rs7168915) | 15 | 78836547 | A/T | *MORF4L1* | intergenic | Novel | Confirmed | 5.97E-04 | 4.04E-02 |
| [rs7678](https://pubs.broadinstitute.org/mammals/haploreg/detail_v4.1.php?query=&id=rs7678) | 22 | 24417197 | T/A | *SPECC1L* | 3'-UTR | Novel | Novel | 9.16E-04 | 4.54E-02 |
| [rs7698460](https://pubs.broadinstitute.org/mammals/haploreg/detail_v4.1.php?query=&id=rs7698460) | 4 | 155745067 | A/T | *RP11-588K22.2* | intergenic | Novel | Novel | 4.23E-06 | 1.16E-02 |
| [rs783147](https://pubs.broadinstitute.org/mammals/haploreg/detail_v4.1.php?query=&id=rs783147) | 6 | 160716958 | A/T | *PLG* | intronic | Novel | Confirmed | 6.14E-07 | 2.13E-03 |
| [rs8025960](https://pubs.broadinstitute.org/mammals/haploreg/detail_v4.1.php?query=&id=rs8025960) | 15 | 78804331 | A/T | *ADAMTS7* | intronic | Novel | Confirmed | 5.09E-07 | 2.09E-03 |
| [rs8039305](https://pubs.broadinstitute.org/mammals/haploreg/detail_v4.1.php?query=&id=rs8039305) | 15 | 90879313 | C/G | *FURIN* | intronic | Novel | Confirmed | 1.33E-15 | 4.55E-11 |
| [rs8105944](https://pubs.broadinstitute.org/mammals/haploreg/detail_v4.1.php?query=&id=rs8105944) | 19 | 51047598 | T/A | *KLK13* | intergenic | Novel | Novel | 3.87E-07 | 1.51E-03 |
| [rs821551](https://pubs.broadinstitute.org/mammals/haploreg/detail_v4.1.php?query=&id=rs821551) | 1 | 155718789 | A/T | *DAP3* | intronic | Novel | Novel | 1.54E-08 | 1.20E-04 |
| [rs866919](https://pubs.broadinstitute.org/mammals/haploreg/detail_v4.1.php?query=&id=rs866919) | 10 | 30224354 | T/A | *RP11-305E6.1* | intergenic | Novel | Novel | 1.77E-06 | 1.83E-03 |
| [rs93139](https://pubs.broadinstitute.org/mammals/haploreg/detail_v4.1.php?query=&id=rs93139) | 11 | 9738061 | C/G | *SWAP70* | intronic | Novel | Confirmed | 3.46E-05 | 2.45E-02 |
| [rs9381462](https://pubs.broadinstitute.org/mammals/haploreg/detail_v4.1.php?query=&id=rs9381462) | 6 | 12873543 | G/C | *PHACTR1* | intronic | Confirmed | Confirmed | 3.14E-05 | 2.96E-02 |
| [rs9515203](https://pubs.broadinstitute.org/mammals/haploreg/detail_v4.1.php?query=&id=rs9515203) | 13 | 110397276 | C/G | *COL4A2* | intronic | Confirmed | Confirmed | 1.98E-04 | 9.93E-03 |
| [rs965098](https://pubs.broadinstitute.org/mammals/haploreg/detail_v4.1.php?query=&id=rs965098) | 21 | 15185306 | A/T | *AF127577.12* | intergenic | Novel | Novel | 1.79E-04 | 4.30E-02 |
| [rs990619](https://pubs.broadinstitute.org/mammals/haploreg/detail_v4.1.php?query=&id=rs990619) | 4 | 155586526 | G/C | *RP13-487K5.1* | intergenic | Novel | Novel | 1.76E-06 | 5.82E-03 |
| [rs998584](https://pubs.broadinstitute.org/mammals/haploreg/detail_v4.1.php?query=&id=rs998584) | 6 | 43790159 | A/T | *VEGFA* | intergenic | Novel | Novel | 1.28E-05 | 2.72E-02 |
