# Supplementary material for: Identification of novel SNPs associated with coronary artery disease and birth weight using a pleiotropic cFDR method
Source: Aging (Albany NY). 2020 Dec 19;13(3):3618–44. doi: 10.18632/aging.202322 (PMC7906162; doi:10.18632/aging.202322)
Supplement: Supplementary Table 2 [file aging-13-202322-s003.pdf]

SUPPLEMENTARY TABLE

Supplementary Table 2. 26 SNPs in high LD (R^2> 0.6) with CAD-associated loci.

SNP	Traits	Proxy SNP	R^2	*P*_value
rs10781976	Coronary artery disease	rs4888378	0.922	6E-15
rs10818580	Coronary artery disease	rs10818576	0.874	8E-09
rs12044531	Coronary artery disease	rs61776719	0.630	1E-09
rs13070927	Cardiovascular disease	rs2131570	0.992	5E-09
rs1418278	Coronary artery disease	rs10826753	0.692	2E-08
rs1541853	Ischemic stroke	rs7582720	1.000	4E-09
rs2001945	Coronary artery disease	rs6982502	0.988	8E-23
rs2166529	Coronary artery disease	rs6743030	0.926	2E-23
rs2238151	Ischemic stroke	rs10744777	0.995	4E-09
rs2306374	Coronary artery disease	rs185244	0.938	2E-17
rs2812	Coronary artery disease	rs9892152	1.000	6E-11
rs34759087	Coronary artery disease (myocardial infarction)	rs7623687	0.714	4E-10
rs3754211	Coronary artery disease	rs6587520	0.750	9E-09
rs4245791	Coronary artery disease (myocardial infarction)	rs4299376	0.968	6E-10
rs4420638	Coronary artery disease	rs56131196	1.000	2E-14
rs4767293	Ischemic stroke	rs10744777	0.946	4E-09
rs4803455	Coronary artery disease	rs2288874	0.741	4E-16
rs583489	Coronary artery disease	rs518594	0.694	1E-12
rs6922782	Ischemic stroke	rs4714955	0.617	4E-11
rs7164299	Coronary artery disease	rs734780	0.960	4E-10
rs7168915	Coronary artery disease (myocardial infarction)	rs7164479	0.731	6E-18
rs7678	Coronary artery disease	rs6004124	0.929	3E-09
rs7698460	Coronary artery disease	rs13131930	0.720	5E-19
rs93139	Coronary artery disease	rs10840293	0.944	9E-13
rs990619	Coronary artery disease	rs1842896	0.984	1E-11
rs998584	Cardiovascular disease	rs6905288	0.688	1E-12
