# Supplementary material for: Identification of novel SNPs associated with coronary artery disease and birth weight using a pleiotropic cFDR method
Source: Aging (Albany NY). 2020 Dec 19;13(3):3618–44. doi: 10.18632/aging.202322 (PMC7906162; doi:10.18632/aging.202322)
Supplement: Supplementary Table 3 [file aging-13-202322-s004.docx]

**Supplementary Table 3.** Conditional FDR values of 111 SNPs for CAD given the BW in validation dataset in validation dataset (cFDR ≤ 0.05).

| **SNP** | **Chr** | **Pos** | **Alt** | **Gene** | **Annotation** | ***P*_value** | **cFDR** | **Validation** |
| --- | --- | --- | --- | --- | --- | --- | --- | --- |
| rs10080815 | 6 | 160266380 | G/C | *SLC22A2* | intergenic | 2.54E-23 | 8.75E-19 | Yes |
| rs10195178 | 2 | 226334547 | G/A | *AC068138.1* | intergenic | 9.26E-05 | 4.69E-02 | No |
| rs1029212 | 6 | 133850341 | G/C | *RP4-662A9.2* | intronic | 3.99E-08 | 2.02E-04 | Yes |
| rs1041602 | 1 | 56547526 | C/T | *PPAP2B* | intronic | 2.22E-08 | 3.57E-05 | No |
| rs10423964 | 19 | 31273945 | C/T | *TSHZ3* | intergenic | 2.38E-05 | 4.18E-02 | No |
| rs1042725 | 12 | 65964567 | C/T | *HMGA2* | 3'-UTR | 5.25E-03 | 1.05E-02 | No |
| rs1047418 | 14 | 75139089 | G/C | *TMED10* | intronic | 1.71E-06 | 3.52E-03 | Yes |
| rs10774625 | 12 | 111472415 | G/C | *ATXN2* | intronic | 9.22E-14 | 5.16E-12 | Yes |
| rs10781976 | 16 | 75280940 | T/A | *U6* | intergenic | 1.61E-05 | 1.38E-02 | Yes |
| rs10818580 | 9 | 121652743 | A/T | *DAB2IP* | intronic | 6.47E-06 | 1.51E-02 | Yes |
| rs10965212 | 9 | 22023796 | A/T | *RP11-145E5.5* | intronic | 3.38E-36 | 1.49E-31 | Yes |
| rs10965228 | 9 | 22082381 | G/C | *CDKN2B-AS1* | intronic | 1.67E-05 | 3.09E-02 | Yes |
| rs11066301 | 12 | 112433568 | G/C | *PTPN11* | intronic | 5.87E-07 | 9.98E-05 | Yes |
| rs11079045 | 17 | 42435651 | A/T | *U7* | intergenic | 6.28E-06 | 1.10E-02 | Yes |
| rs11100902 | 4 | 145874074 | A/G | *ZNF827* | intronic | 3.06E-05 | 4.23E-02 | No |
| rs11204725 | 1 | 150770224 | T/C | *CTSS* | intergenic | 1.49E-05 | 1.32E-02 | No |
| rs11206803 | 1 | 56411837 | T/A | *RP4-710M16.2* | intergenic | 2.10E-05 | 4.86E-03 | Yes |
| rs11238956 | 10 | 44254406 | C/G | *RP11-20J15.2* | intergenic | 2.53E-05 | 3.86E-02 | Yes |
| rs11591147 | 1 | 55039974 | T/A | *PCSK9* | missense | 2.84E-10 | 2.64E-06 | Yes |
| rs11617955 | 13 | 110165755 | A/T | *COL4A1* | intronic | 4.14E-10 | 3.21E-06 | Yes |
| rs11668477 | 19 | 11084354 | G/C | *LDLR* | intergenic | 1.29E-12 | 1.09E-08 | Yes |
| rs11675251 | 2 | 203384676 | G/C | *ABI2* | intronic | 5.83E-06 | 1.55E-02 | Yes |
| rs11853441 | 15 | 90856978 | T/C | *Metazoa_SRP* | intergenic | 1.66E-04 | 9.38E-03 | No |
| rs12044531 | 1 | 37987369 | A/T | *SF3A3* | intronic | 9.36E-06 | 1.85E-02 | Yes |
| rs12306172 | 12 | 54145221 | A/T | *RP11-834C11.11* | intergenic | 1.12E-04 | 2.20E-03 | Yes |
| rs12438008 | 15 | 78792338 | G/A | *ADAMTS7* | intronic | 3.73E-10 | 3.09E-06 | No |
| rs12530920 | 7 | 19016174 | C/G | *TWIST1* | intergenic | 1.70E-05 | 1.45E-02 | Yes |
| rs12691693 | 2 | 144497643 | T/C | *ZEB2* | intronic | 5.79E-05 | 3.89E-02 | No |
| rs12792912 | 11 | 102930574 | G/C | *MMP13* | intergenic | 3.22E-05 | 1.55E-02 | Yes |
| rs12891473 | 14 | 34983051 | T/C | *SRP54* | 5'-UTR | 2.33E-05 | 3.74E-02 | No |
| rs13265868 | 8 | 20085516 | G/A | *AC100802.3* | intergenic | 1.59E-05 | 2.99E-02 | No |
| rs1333050 | 9 | 22125914 | T/A | *CDKN2B-AS1* | intergenic | 2.39E-41 | 4.09E-37 | Yes |
| rs13382133 | 19 | 17748127 | T/A | *FCHO1* | intronic | 2.44E-05 | 1.43E-02 | Yes |
| rs1418278 | 10 | 30009149 | G/C | *KIAA1462* | intergenic | 3.28E-09 | 3.84E-06 | Yes |
| rs1482472 | 10 | 44187525 | C/G | *RP11-20J15.2* | intergenic | 2.00E-06 | 6.07E-03 | Yes |
| rs1531837 | 11 | 103797882 | A/G | *RP11* | intergenic | 5.93E-06 | 1.36E-02 | No |
| rs1541853 | 2 | 202970454 | C/G | *WDR12* | intronic | 6.58E-19 | 1.67E-14 | Yes |
| rs1547705 | 9 | 22082376 | C/G | *CDKN2B-AS1* | intronic | 1.73E-12 | 1.80E-08 | Yes |
| rs16891156 | 6 | 160187772 | C/G | *SLC22A2* | intergenic | 3.00E-10 | 1.66E-06 | Yes |
| rs16986953 | 2 | 19742712 | A/T | *AC019055.1* | intergenic | 4.77E-10 | 2.56E-06 | Yes |
| rs17244648 | 15 | 78910407 | C/T | *CTSH* | intergenic | 9.15E-07 | 2.49E-03 | No |
| rs17477113 | 2 | 203501730 | G/C | *RAPH1* | intronic | 7.61E-06 | 1.78E-02 | Yes |
| rs17726488 | 10 | 72158300 | C/T | *ASCC1* | intronic | 1.01E-05 | 1.87E-02 | No |
| rs2001945 | 8 | 125465736 | C/G | *RP11-136O12.2* | intergenic | 7.64E-07 | 8.01E-04 | Yes |
| rs2011559 | 2 | 205449947 | A/G | *PARD3B* | intronic | 2.44E-05 | 3.20E-02 | No |
| rs2126202 | 17 | 2192418 | A/T | *SMG6* | intronic | 3.27E-05 | 2.76E-02 | Yes |
| rs2144723 | 6 | 160700358 | T/A | *RP1-81D8.3* | intergenic | 1.37E-09 | 7.03E-06 | Yes |
| rs2146238 | 14 | 99706392 | G/C | *CYP46A1* | intronic | 1.14E-05 | 2.49E-02 | Yes |
| rs2161969 | 2 | 217815847 | G/A | *TNS1* | intronic | 2.31E-05 | 3.88E-02 | No |
| rs2166529 | 2 | 85515052 | T/A | *Metazoa_SRP* | intergenic | 4.49E-12 | 3.52E-08 | Yes |
| rs2238151 | 12 | 111774029 | C/G | *ALDH2* | intronic | 4.29E-05 | 2.26E-02 | Yes |
| rs2243621 | 6 | 31464043 | C/T | *HCP5* | 3'-UTR | 2.04E-04 | 2.56E-02 | No |
| rs2327430 | 6 | 133888899 | T/A | *TCF21* | intergenic | 1.40E-05 | 2.80E-02 | Yes |
| rs2339940 | 2 | 24028917 | T/A | *MFSD2B* | intergenic | 3.61E-03 | 4.45E-02 | Yes |
| rs2347252 | 3 | 138376683 | T/C | *MRAS* | intronic | 1.42E-09 | 6.56E-06 | No |
| rs251023 | 5 | 141513843 | A/G | *PCDHGA8* | intergenic | 8.34E-06 | 2.05E-02 | No |
| rs268 | 8 | 19956018 | A/G | *LPL* | missense | 2.38E-05 | 4.33E-02 | No |
| rs2709437 | 2 | 5983360 | T/C | *AC073479.1* | intronic | 4.38E-05 | 4.65E-02 | No |
| rs2802490 | 10 | 44103565 | A/T | *AL512640.1* | intergenic | 7.19E-09 | 2.89E-05 | Yes |
| rs2812 | 17 | 64323758 | C/G | *TEX2* | 3'-UTR | 1.77E-06 | 3.22E-03 | Yes |
| rs2836631 | 21 | 38693982 | G/C | *ERG* | intergenic | 6.17E-05 | 4.62E-02 | Yes |
| rs2971671 | 7 | 44171738 | T/C | *GCK* | intronic | 2.83E-05 | 4.15E-02 | No |
| rs3172494 | 3 | 48694054 | T/A | *IP6K2* | 3'-UTR | 1.40E-07 | 5.18E-04 | Yes |
| rs3751395 | 13 | 28384818 | A/T | *FLT1* | intronic | 3.97E-07 | 5.36E-04 | Yes |
| rs3754211 | 1 | 150979381 | A/T | *ANXA9* | intergenic | 8.99E-06 | 9.16E-03 | Yes |
| rs3783106 | 13 | 110189806 | T/C | *COL4A1* | intronic | 2.97E-05 | 3.11E-02 | No |
| rs3869098 | 6 | 31034675 | A/G | *MUC22* | missense | 2.99E-06 | 7.07E-03 | No |
| rs3918291 | 6 | 160407110 | C/G | *SLC22A3* | synonymous | 1.36E-06 | 4.41E-03 | Yes |
| rs405509 | 19 | 44905579 | G/C | *APOE* | intergenic | 3.50E-07 | 1.10E-03 | Yes |
| rs4233701 | 2 | 23706216 | C/G | *KLHL29* | intronic | 5.92E-03 | 4.50E-02 | Yes |
| rs4245791 | 2 | 43847292 | T/A | *ABCG8* | intronic | 8.57E-10 | 3.85E-06 | Yes |
| rs4290163 | 10 | 102851169 | T/A | *C10orf32* | intergenic | 2.00E-05 | 1.19E-02 | Yes |
| rs445925 | 19 | 44912383 | A/T | *APOC1* | intergenic | 1.19E-12 | 1.76E-08 | Yes |
| rs4481859 | 1 | 222559314 | G/C | *TAF1A* | intronic | 4.36E-07 | 1.54E-03 | Yes |
| rs4678408 | 3 | 138334345 | A/G | *NME9* | intergenic | 4.71E-06 | 1.14E-02 | No |
| rs4704942 | 5 | 158466352 | G/A | *RP11* | intergenic | 8.83E-03 | 4.95E-02 | No |
| rs4760 | 19 | 43648948 | A/G | *PLAUR* | missense | 2.56E-05 | 1.33E-02 | No |
| rs4767293 | 12 | 112025492 | G/C | *NAA25* | intergenic | 2.70E-06 | 3.79E-03 | Yes |
| rs4773144 | 13 | 110308365 | G/C | *COL4A2* | intronic | 2.81E-05 | 4.21E-02 | Yes |
| rs4803455 | 19 | 41345604 | A/T | *TGFB1* | intronic | 6.06E-07 | 1.69E-03 | Yes |
| rs4888432 | 16 | 75477075 | A/G | *RP11* | 3'-UTR | 3.93E-05 | 2.40E-02 | No |
| rs583489 | 10 | 44243240 | G/C | *RP11-20J15.2* | intergenic | 7.34E-11 | 6.93E-07 | Yes |
| rs6016377 | 20 | 40544088 | C/T | *SNORD112* | intergenic | 1.97E-03 | 2.95E-02 | No |
| rs624249 | 6 | 160258368 | A/T | *SLC22A2* | synonymous | 6.31E-10 | 4.50E-06 | Yes |
| rs629001 | 1 | 109296296 | C/T | *MYBPHL* | missense | 1.84E-07 | 6.77E-04 | No |
| rs630014 | 9 | 133274306 | G/C | *ABO* | intronic | 1.74E-05 | 2.05E-03 | Yes |
| rs6492260 | 13 | 110308889 | G/A | *COL4A2* | intronic | 2.11E-05 | 3.79E-02 | No |
| rs650985 | 1 | 109658958 | C/T | *GSTM4* | intronic | 4.58E-06 | 6.48E-03 | No |
| rs6546693 | 2 | 71453502 | T/C | *DYSF* | intergenic | 1.13E-04 | 4.60E-02 | No |
| rs655246 | 1 | 109289661 | G/C | *MYBPHL* | intergenic | 3.47E-06 | 7.97E-03 | Yes |
| rs656461 | 6 | 149477699 | T/A | *ZC3H12D* | intronic | 3.45E-05 | 4.06E-02 | Yes |
| rs6673081 | 1 | 155017119 | C/G | *ZBTB7B* | 3'-UTR | 6.58E-03 | 3.73E-02 | Yes |
| rs6713510 | 2 | 226169783 | A/T | *AC068138.1* | intronic | 1.13E-04 | 1.07E-02 | Yes |
| rs6859 | 19 | 44878777 | A/G | *PVRL2* | 3'-UTR | 3.39E-06 | 4.32E-03 | No |
| rs688359 | 6 | 160044259 | A/T | *IGF2R* | intronic | 2.93E-06 | 2.95E-03 | Yes |
| rs6922782 | 6 | 12948156 | A/T | *PHACTR1* | intronic | 1.06E-15 | 2.54E-11 | Yes |
| rs7164299 | 15 | 89036690 | C/G | *RP11-326A19.4* | intergenic | 3.34E-07 | 1.23E-03 | Yes |
| rs7168915 | 15 | 78836547 | A/T | *MORF4L1* | intergenic | 9.11E-12 | 8.05E-08 | Yes |
| rs744910 | 15 | 67154447 | G/A | *SMAD3* | intronic | 1.98E-05 | 1.28E-02 | No |
| rs760336 | 10 | 122479194 | T/C | *HTRA1* | intronic | 1.14E-04 | 4.88E-02 | No |
| rs7678 | 22 | 24417197 | T/A | *SPECC1L* | 3'-UTR | 4.75E-06 | 9.38E-03 | Yes |
| rs7698460 | 4 | 155745067 | A/T | *RP11-588K22.2* | intergenic | 2.40E-05 | 2.14E-02 | Yes |
| rs783147 | 6 | 160716958 | A/T | *PLG* | intronic | 4.09E-07 | 1.04E-03 | Yes |
| rs8039305 | 15 | 90879313 | C/G | *FURIN* | intronic | 1.06E-12 | 2.86E-11 | Yes |
| rs8191855 | 6 | 160064028 | G/A | *IGF2R* | intronic | 6.41E-08 | 2.31E-04 | No |
| rs866919 | 10 | 30224354 | T/A | *RP11-305E6.1* | intergenic | 5.13E-04 | 3.92E-02 | Yes |
| rs93139 | 11 | 9738061 | C/G | *SWAP70* | intronic | 4.62E-08 | 1.58E-04 | Yes |
| rs9381462 | 6 | 12873543 | G/C | *PHACTR1* | intronic | 1.42E-13 | 1.37E-09 | Yes |
| rs9515203 | 13 | 110397276 | C/G | *COL4A2* | intronic | 6.48E-10 | 4.26E-06 | Yes |
| rs965098 | 21 | 15185306 | A/T | *AF127577.12* | intergenic | 5.75E-04 | 4.84E-02 | Yes |
| rs9659073 | 1 | 150554878 | G/A | *RP11* | intronic | 2.02E-05 | 3.01E-02 | No |
