# Supplementary material for: Identification of novel SNPs associated with coronary artery disease and birth weight using a pleiotropic cFDR method
Source: Aging (Albany NY). 2020 Dec 19;13(3):3618–44. doi: 10.18632/aging.202322 (PMC7906162; doi:10.18632/aging.202322)
Supplement: Supplementary Table 4 [file aging-13-202322-s005.docx]

**Supplementary Table 4.** Conditional FDR values of 203 SNPs for BW given the CAD (cFDR ≤ 0.05).

| **SNP** | **Chr** | **Pos** | **Alt** | **Gene** | **Annotation** | **SNP Type** | **Gene Type** | ***P*_value** | **cFDR** |
| --- | --- | --- | --- | --- | --- | --- | --- | --- | --- |
| rs10008032 | 4 | 38743861 | C/G | *RN5S158* | intergenic | Novel | Novel | 3.70E-05 | 1.02E-02 |
| rs1003573 | 7 | 44223857 | C/G | *CAMK2B* | intronic | Novel | Novel | 1.60E-05 | 1.99E-02 |
| rs10049090 | 3 | 157079913 | A/T | *RP11-6F2.4* | intergenic | Novel | Novel | 2.80E-42 | 5.86E-38 |
| rs1012626 | 6 | 20577330 | T/A | *CDKAL1* | intronic | Novel | Confirmed | 4.50E-15 | 1.88E-11 |
| rs1012635 | 6 | 20675064 | G/C | *CDKAL1* | intronic | Confirmed | Confirmed | 4.00E-10 | 2.95E-06 |
| rs10130093 | 14 | 89332778 | A/T | *FOXN3* | intronic | Novel | Novel | 3.30E-06 | 5.93E-03 |
| rs10221235 | 17 | 70097771 | C/G | *KCNJ16* | intronic | Novel | Novel | 4.10E-05 | 3.21E-02 |
| rs10283100 | 8 | 119583783 | G/C | *ENPP2* | missense | Novel | Novel | 1.80E-05 | 9.79E-03 |
| rs1042725 | 12 | 65964567 | T/A | *HMGA2* | 3'-UTR | Novel | Confirmed | 7.10E-32 | 2.92E-28 |
| rs1044299 | 1 | 176842737 | T/A | *PAPPA2* | 3'-UTR | Novel | Novel | 3.60E-06 | 7.53E-03 |
| rs10457487 | 6 | 127198089 | A/T | *RP11-73O6.4* | intergenic | Novel | Novel | 3.90E-05 | 2.02E-02 |
| rs10461018 | 3 | 46953752 | T/A | *CCDC12* | intronic | Novel | Novel | 2.70E-05 | 3.26E-02 |
| rs10494967 | 1 | 213828251 | A/T | *PROX1-AS1* | intronic | Novel | Novel | 5.40E-05 | 3.62E-02 |
| rs10514019 | 18 | 71669759 | C/G | *RP11-723G8.2* | intergenic | Novel | Novel | 1.40E-05 | 1.94E-02 |
| rs10520597 | 15 | 85627024 | A/T | *RP11-815J21.3* | intronic | Novel | Novel | 1.80E-04 | 4.11E-02 |
| rs10774625 | 12 | 111472415 | G/C | *ATXN2* | intronic | Novel | Novel | 3.40E-06 | 3.06E-05 |
| rs10786156 | 10 | 94254865 | G/C | *PLCE1* | intronic | Novel | Novel | 1.80E-07 | 5.95E-05 |
| rs10786706 | 10 | 102740902 | T/A | *SFXN2* | intergenic | Novel | Novel | 1.10E-03 | 3.17E-02 |
| rs10804733 | 3 | 148913142 | G/C | *RP11-680B3.2* | intergenic | Novel | Novel | 8.70E-05 | 1.69E-02 |
| rs10840346 | 11 | 10041452 | A/T | *SBF2* | intronic | Novel | Novel | 7.00E-07 | 3.39E-04 |
| rs10878353 | 12 | 65988752 | C/G | *HMGA2* | intergenic | Novel | Confirmed | 1.40E-08 | 6.04E-05 |
| rs10946403 | 6 | 20717173 | G/C | *CDKAL1* | intronic | Novel | Confirmed | 3.90E-17 | 2.16E-13 |
| rs11066301 | 12 | 112433568 | G/C | *PTPN11* | intronic | Novel | Novel | 7.80E-04 | 6.50E-03 |
| rs11079803 | 17 | 47942535 | A/T | *PNPO* | intronic | Novel | Novel | 9.70E-06 | 1.26E-02 |
| rs11090046 | 22 | 41373079 | C/G | *TEF* | intronic | Novel | Novel | 4.70E-06 | 7.64E-03 |
| rs11125079 | 2 | 46505076 | T/A | *ATP6V1E2* | intergenic | Novel | Novel | 9.50E-06 | 5.58E-03 |
| rs11172113 | 12 | 57133500 | C/G | *LRP1* | intronic | Novel | Novel | 1.90E-03 | 3.18E-02 |
| rs11175992 | 12 | 65997616 | A/T | *RP11-366L20.4* | intergenic | Novel | Novel | 5.40E-06 | 5.98E-03 |
| rs11187076 | 10 | 92577206 | A/T | *IDE* | intergenic | Novel | Confirmed | 8.10E-08 | 1.02E-04 |
| rs11206803 | 1 | 56411837 | T/A | *RP4-710M16.2* | intergenic | Novel | Novel | 3.00E-03 | 3.51E-02 |
| rs1147322 | 9 | 122913322 | G/C | *ZBTB6* | 5'-UTR | Novel | Novel | 9.90E-11 | 1.09E-06 |
| rs11690295 | 2 | 111893739 | C/G | *MERTK* | intergenic | Novel | Novel | 1.90E-06 | 2.88E-03 |
| rs11853441 | 15 | 90856978 | G/C | *Metazoa_SRP* | intergenic | Novel | Novel | 1.70E-04 | 3.01E-02 |
| rs11889485 | 2 | 46257398 | A/T | *EPAS1* | intergenic | Novel | Confirmed | 2.00E-13 | 2.22E-09 |
| rs1202427 | 7 | 149240045 | A/T | *ZNF212* | intronic | Novel | Novel | 4.00E-06 | 5.84E-03 |
| rs1206028 | 19 | 42351015 | C/G | *MEGF8* | intronic | Novel | Novel | 3.90E-05 | 3.46E-02 |
| rs12148530 | 15 | 96542056 | C/G | *7SK* | intergenic | Novel | Novel | 6.30E-04 | 1.64E-02 |
| rs1218565 | 1 | 154845211 | T/A | *KCNN3* | intronic | Novel | Novel | 2.90E-06 | 2.11E-03 |
| rs12269934 | 11 | 58386843 | C/G | *OR5B3* | intergenic | Novel | Novel | 2.20E-05 | 3.00E-02 |
| rs12281432 | 11 | 10235654 | A/T | *SBF2* | intronic | Novel | Novel | 1.10E-05 | 1.60E-02 |
| rs12306172 | 12 | 54145221 | A/T | *RP11-834C11.11* | intergenic | Novel | Novel | 3.80E-06 | 8.89E-05 |
| rs12359055 | 10 | 24790442 | T/A | *PRTFDC1* | intergenic | Novel | Novel | 3.00E-04 | 2.78E-02 |
| rs12371967 | 12 | 65952934 | C/G | *HMGA2* | intronic | Novel | Confirmed | 2.20E-10 | 7.51E-07 |
| rs12476224 | 2 | 56154789 | G/C | *AC007743.1* | intergenic | Novel | Novel | 2.40E-04 | 4.59E-02 |
| rs12536475 | 7 | 35284979 | A/T | *AC009531.2* | intergenic | Novel | Novel | 2.20E-05 | 3.93E-03 |
| rs12623454 | 2 | 120568721 | C/G | *AC073257.1* | intergenic | Novel | Novel | 2.40E-05 | 1.06E-02 |
| rs12643660 | 4 | 105465603 | G/C | *PPA2* | intronic | Novel | Novel | 3.50E-05 | 2.59E-02 |
| rs12656216 | 5 | 36160566 | A/T | *SKP2* | intronic | Novel | Novel | 2.00E-06 | 5.15E-03 |
| rs12828089 | 12 | 46210774 | A/T | *SLC38A1* | intronic | Novel | Novel | 3.70E-05 | 3.43E-02 |
| rs12865243 | 13 | 40104683 | A/T | *LINC00332* | intergenic | Novel | Confirmed | 3.00E-06 | 7.69E-03 |
| rs13035774 | 2 | 24135782 | T/A | *AC008073.6* | intronic | Novel | Novel | 2.40E-04 | 4.35E-03 |
| rs1319046 | 15 | 98626817 | T/A | *RP11-35O15.1* | intergenic | Novel | Novel | 6.40E-06 | 8.98E-03 |
| rs1319859 | 15 | 98687034 | A/T | *IGF1R* | intronic | Novel | Confirmed | 5.00E-04 | 4.11E-02 |
| rs1319869 | 15 | 98669256 | T/A | *IGF1R* | intronic | Novel | Confirmed | 7.40E-06 | 1.85E-04 |
| rs1319888 | 17 | 49469749 | C/G | *RP11-81K2.1* | intergenic | Novel | Novel | 3.00E-05 | 3.34E-02 |
| rs1361024 | 6 | 151749793 | A/T | *ESR1* | intronic | Novel | Confirmed | 8.00E-06 | 7.68E-03 |
| rs1384539 | 3 | 157094152 | A/T | *RP11-6F2.4* | intronic | Novel | Novel | 2.30E-05 | 1.55E-02 |
| rs1389923 | 6 | 165747283 | T/A | *RP11-252P19.3* | intergenic | Novel | Novel | 1.50E-05 | 6.03E-03 |
| rs1415181 | 1 | 214857808 | C/G | *KCNK2* | intergenic | Novel | Novel | 3.70E-06 | 6.61E-03 |
| rs1415701 | 6 | 130024690 | A/T | *L3MBTL3* | intronic | Confirmed | Confirmed | 4.00E-11 | 9.76E-08 |
| rs1425661 | 4 | 67193004 | C/G | *RP11-584P21.2* | intergenic | Novel | Novel | 2.80E-05 | 2.11E-02 |
| rs143384 | 20 | 35437976 | G/C | *GDF5* | 5'-UTR | Novel | Novel | 6.40E-07 | 5.89E-04 |
| rs1451156 | 2 | 46605463 | T/A | *PIGF* | intronic | Novel | Novel | 2.60E-05 | 3.00E-02 |
| rs1475643 | 10 | 102786710 | T/A | *WBP1L* | intronic | Novel | Novel | 1.50E-04 | 3.27E-02 |
| rs1480933 | 4 | 119512093 | T/A | *PDE5A* | intronic | Novel | Novel | 2.20E-03 | 3.52E-02 |
| rs1488691 | 1 | 91867308 | G/C | *TGFBR3* | intronic | Novel | Novel | 1.40E-05 | 7.89E-03 |
| rs149514 | 5 | 65555910 | A/T | *CENPK* | intronic | Novel | Novel | 8.30E-05 | 3.34E-02 |
| rs1547669 | 6 | 33807864 | G/C | *MLN* | intergenic | Novel | Novel | 1.00E-05 | 1.57E-02 |
| rs1548304 | 22 | 42295232 | T/A | *TCF20* | intergenic | Novel | Novel | 6.90E-06 | 1.17E-02 |
| rs1580278 | 4 | 103219691 | A/T | *CENPE* | intergenic | Novel | Novel | 3.10E-05 | 2.63E-02 |
| rs1638410 | 10 | 116766015 | C/G | *HSPA12A* | intergenic | Novel | Novel | 5.70E-05 | 1.38E-02 |
| rs16887484 | 5 | 57859481 | G/C | *AC116606.1* | intergenic | Novel | Novel | 3.20E-05 | 1.89E-02 |
| rs16952999 | 18 | 936560 | T/A | *RP11-672L10.1* | intergenic | Novel | Novel | 4.30E-05 | 1.12E-02 |
| rs17015692 | 4 | 89502952 | T/A | *RP11-115D19.1* | intergenic | Novel | Novel | 2.80E-05 | 2.13E-02 |
| rs17290714 | 3 | 47854215 | T/A | *MAP4* | intronic | Novel | Novel | 1.80E-05 | 1.02E-02 |
| rs17384555 | 18 | 5618174 | T/A | *EPB41L3* | intergenic | Novel | Novel | 2.30E-05 | 2.89E-02 |
| rs17472967 | 4 | 78704724 | A/T | *RP11-792D21.2* | intergenic | Novel | Novel | 1.10E-04 | 4.30E-02 |
| rs17566087 | 10 | 69219200 | G/C | *RP11-227H15.4* | intergenic | Novel | Novel | 2.30E-05 | 2.17E-03 |
| rs17647531 | 3 | 48138448 | A/T | *CDC25A* | intergenic | Novel | Novel | 7.40E-05 | 3.77E-02 |
| rs17745230 | 2 | 46263191 | T/A | *EPAS1* | intergenic | Novel | Confirmed | 9.10E-07 | 1.50E-03 |
| rs17767418 | 17 | 30479276 | T/A | *GOSR1* | intronic | Novel | Novel | 2.60E-08 | 3.66E-05 |
| rs17826255 | 17 | 31006498 | C/G | *RNF135* | intronic | Novel | Novel | 2.80E-05 | 1.69E-02 |
| rs1797081 | 10 | 16832566 | C/G | *CUBN* | intronic | Novel | Novel | 1.50E-05 | 1.91E-02 |
| rs1861044 | 4 | 15537875 | G/C | *CC2D2A* | intronic | Novel | Novel | 1.90E-03 | 3.25E-02 |
| rs1893197 | 18 | 5618387 | T/A | *EPB41L3* | intergenic | Novel | Novel | 3.70E-05 | 3.71E-02 |
| rs1983127 | 10 | 69230145 | T/A | *RP11-227H15.4* | intronic | Novel | Novel | 3.70E-05 | 3.09E-02 |
| rs2013116 | 12 | 92502603 | T/A | *RP11-693J15.4* | intergenic | Novel | Novel | 9.70E-05 | 2.31E-02 |
| rs2087826 | 4 | 144718166 | A/T | *HHIP* | intronic | Novel | Confirmed | 4.00E-09 | 1.91E-05 |
| rs2150052 | 9 | 111182787 | T/A | *RP11-202G18.1* | intergenic | Confirmed | Novel | 2.80E-08 | 5.16E-05 |
| rs2160875 | 12 | 4418156 | T/A | *FGF6* | intergenic | Novel | Novel | 1.30E-04 | 4.02E-02 |
| rs2191883 | 7 | 35233679 | C/G | *TBX20* | intronic | Novel | Confirmed | 4.20E-06 | 8.99E-04 |
| rs2214409 | 4 | 105111788 | T/A | *RP11-556I14.1* | intergenic | Novel | Novel | 6.00E-06 | 6.10E-03 |
| rs222837 | 17 | 7229237 | T/A | *DVL2* | synonymous | Novel | Novel | 3.90E-07 | 7.93E-04 |
| rs2268310 | 7 | 44637499 | T/A | *OGDH* | intronic | Novel | Novel | 2.00E-03 | 3.10E-02 |
| rs2288291 | 11 | 12474060 | C/G | *PARVA* | intronic | Novel | Novel | 4.60E-05 | 2.51E-02 |
| rs2290228 | 7 | 128748594 | A/T | *CALU* | missense | Novel | Novel | 1.90E-04 | 4.94E-02 |
| rs2296742 | 6 | 33692016 | A/T | *ITPR3* | intronic | Novel | Novel | 1.40E-04 | 3.06E-02 |
| rs2298229 | 13 | 53028835 | G/C | *OLFM4* | 5'-UTR | Novel | Novel | 4.00E-05 | 4.10E-02 |
| rs2306531 | 3 | 157099873 | T/A | *RP11-6F2.4* | intronic | Novel | Novel | 8.60E-06 | 1.64E-02 |
| rs2339940 | 2 | 24028917 | T/A | *MFSD2B* | intergenic | Novel | Novel | 4.50E-07 | 1.67E-05 |
| rs2423512 | 20 | 10705728 | C/G | *JAG1* | intergenic | Novel | Confirmed | 2.10E-07 | 2.39E-04 |
| rs2493995 | 6 | 20753888 | A/T | *CDKAL1* | intronic | Novel | Confirmed | 2.80E-05 | 2.91E-02 |
| rs2497304 | 10 | 92732959 | T/A | *Y_RNA* | intergenic | Novel | Novel | 1.40E-14 | 4.86E-11 |
| rs2505126 | 10 | 30100897 | A/T | *KIAA1462* | intergenic | Novel | Novel | 2.10E-04 | 1.82E-02 |
| rs2540074 | 9 | 123209217 | C/G | *STRBP* | intronic | Novel | Confirmed | 1.90E-10 | 2.00E-06 |
| rs268691 | 19 | 40443765 | C/G | *SERTAD3* | intronic | Novel | Novel | 4.20E-05 | 3.72E-02 |
| rs2715878 | 2 | 9375405 | G/C | *ASAP2* | intronic | Novel | Novel | 4.70E-05 | 3.66E-02 |
| rs2782980 | 10 | 114021768 | C/G | *ADRB1* | intergenic | Confirmed | Confirmed | 2.50E-05 | 1.16E-02 |
| rs2823025 | 21 | 15052100 | G/C | *AF127577.1* | 3'-UTR | Novel | Novel | 7.90E-07 | 8.11E-04 |
| rs2848465 | 18 | 11871031 | G/C | *GNAL* | intronic | Novel | Novel | 1.80E-04 | 4.86E-02 |
| rs28536742 | 9 | 95520811 | T/A | *PTCH1* | intergenic | Novel | Confirmed | 2.10E-11 | 9.93E-08 |
| rs2886070 | 1 | 156004180 | A/T | *RP11-336K24.4* | intergenic | Novel | Novel | 3.50E-08 | 6.01E-05 |
| rs3198697 | 16 | 15036083 | T/A | *PDXDC1* | synonymous | Novel | Novel | 3.50E-05 | 7.64E-03 |
| rs328890 | 7 | 34973837 | A/T | *DPY19L1* | intronic | Novel | Novel | 2.10E-04 | 4.15E-02 |
| rs32902 | 5 | 77013087 | G/C | *AGGF1* | intergenic | Novel | Novel | 2.80E-05 | 1.90E-02 |
| rs3756668 | 5 | 68300260 | A/T | *PIK3R1* | 3'-UTR | Novel | Novel | 1.70E-05 | 6.91E-04 |
| rs3772587 | 3 | 148859129 | T/A | *CPB1* | intronic | Novel | Confirmed | 1.50E-06 | 1.25E-03 |
| rs3795521 | 1 | 214641228 | T/A | *CENPF* | synonymous | Novel | Novel | 3.00E-06 | 5.84E-03 |
| rs3849774 | 5 | 39437520 | G/C | *DAB2* | intergenic | Novel | Novel | 5.80E-05 | 4.85E-02 |
| rs4143341 | 7 | 159262263 | G/C | *VIPR2* | intergenic | Novel | Novel | 3.20E-06 | 2.07E-03 |
| rs4144650 | 6 | 20938690 | A/T | *CDKAL1* | intronic | Novel | Confirmed | 6.70E-08 | 1.07E-04 |
| rs4233701 | 2 | 23706216 | C/G | *KLHL29* | intronic | Novel | Novel | 5.20E-07 | 2.81E-05 |
| rs4428060 | 2 | 46263662 | T/A | *EPAS1* | intergenic | Novel | Confirmed | 1.70E-09 | 6.58E-06 |
| rs4643791 | 4 | 119344464 | A/T | *FABP2* | intergenic | Novel | Novel | 1.40E-03 | 2.75E-02 |
| rs4677887 | 3 | 123381376 | G/C | *ADCY5* | intronic | Confirmed | Confirmed | 2.30E-12 | 2.55E-08 |
| rs4699908 | 5 | 57618434 | A/T | *CTD-2023N9.3* | intergenic | Novel | Novel | 4.50E-06 | 1.01E-02 |
| rs4704942 | 5 | 158466352 | C/G | *RP11-542A14.1* | intergenic | Novel | Novel | 6.10E-08 | 6.38E-05 |
| rs4710945 | 6 | 20744843 | T/A | *CDKAL1* | intronic | Novel | Confirmed | 9.20E-10 | 1.61E-06 |
| rs4712542 | 6 | 20772587 | T/A | *RP3-348I23.2* | intronic | Novel | Novel | 2.90E-05 | 2.22E-02 |
| rs475931 | 11 | 104914727 | G/C | *CASP12* | intronic | Novel | Novel | 3.60E-05 | 3.70E-02 |
| rs4762119 | 12 | 65770055 | T/A | *HMGA2* | intronic | Novel | Confirmed | 1.10E-05 | 4.32E-03 |
| rs4793636 | 17 | 50062136 | A/T | *ITGA3* | intronic | Novel | Novel | 4.10E-05 | 4.06E-02 |
| rs4796392 | 17 | 7271578 | C/G | *Y_RNA* | intergenic | Novel | Novel | 2.70E-04 | 4.39E-02 |
| rs4812493 | 20 | 41320745 | T/A | *ZHX3* | intergenic | Novel | Novel | 8.20E-06 | 2.13E-03 |
| rs4853831 | 2 | 1809892 | C/G | *MYT1L* | intronic | Novel | Novel | 5.10E-05 | 4.18E-02 |
| rs4875812 | 8 | 1811300 | G/C | *MIR596* | intergenic | Novel | Novel | 4.60E-05 | 1.05E-02 |
| rs4953337 | 2 | 46280709 | G/C | *EPAS1* | intergenic | Novel | Confirmed | 2.10E-04 | 4.46E-02 |
| rs502467 | 3 | 172009573 | C/G | *FNDC3B* | intergenic | Novel | Novel | 1.40E-03 | 2.22E-02 |
| rs533318 | 19 | 40161784 | C/G | *MAP3K10* | intergenic | Novel | Novel | 1.80E-04 | 3.64E-02 |
| rs5742915 | 15 | 74044292 | C/G | *PML* | missense | Novel | Novel | 3.10E-05 | 2.69E-02 |
| rs5765273 | 22 | 45352071 | G/C | *SMC1B* | intronic | Novel | Novel | 9.30E-06 | 8.33E-03 |
| rs6007030 | 22 | 45434479 | G/C | *RP1-102D24.5* | intergenic | Novel | Novel | 2.50E-05 | 2.39E-02 |
| rs6016377 | 20 | 40544088 | T/A | *SNORD112* | intergenic | Confirmed | Novel | 3.60E-10 | 4.32E-07 |
| rs6057610 | 20 | 32653587 | C/G | *C20orf203* | intergenic | Novel | Confirmed | 3.00E-10 | 2.05E-06 |
| rs6072263 | 20 | 41076906 | C/G | *TOP1* | intronic | Novel | Novel | 1.10E-05 | 3.11E-03 |
| rs6075924 | 20 | 22531891 | C/G | *RP11-216C10.1* | intergenic | Novel | Novel | 4.80E-06 | 4.38E-03 |
| rs611003 | 11 | 69630516 | A/T | *CCND1* | intergenic | Novel | Novel | 7.10E-06 | 9.20E-04 |
| rs630014 | 9 | 133274306 | G/C | *ABO* | intronic | Novel | Novel | 4.10E-04 | 1.16E-02 |
| rs6424243 | 1 | 232632402 | A/T | *U6* | intergenic | Novel | Novel | 1.10E-05 | 9.34E-03 |
| rs6437106 | 2 | 157553684 | T/A | *ACVR1C* | intronic | Novel | Novel | 2.70E-05 | 2.99E-02 |
| rs646596 | 1 | 209826803 | C/G | *DIEXF* | intergenic | Novel | Novel | 9.60E-05 | 2.81E-02 |
| rs6484487 | 11 | 30511445 | C/G | *MPPED2* | intronic | Novel | Novel | 1.60E-05 | 9.83E-03 |
| rs6511689 | 19 | 10210413 | C/G | *S1PR2* | intergenic | Novel | Novel | 2.90E-05 | 2.52E-02 |
| rs663344 | 9 | 95554049 | C/G | *PTCH1* | intergenic | Novel | Confirmed | 2.90E-05 | 2.23E-02 |
| rs6673081 | 1 | 155017119 | C/G | *ZBTB7B* | 3'-UTR | Novel | Confirmed | 5.30E-10 | 4.66E-08 |
| rs6700896 | 1 | 65624099 | T/A | *LEPR* | intronic | Novel | Novel | 3.60E-05 | 2.96E-02 |
| rs670950 | 19 | 43777410 | C/G | *KCNN4* | intronic | Novel | Novel | 2.30E-04 | 7.91E-03 |
| rs6713510 | 2 | 226169783 | A/T | *AC068138.1* | intronic | Novel | Novel | 6.70E-04 | 1.29E-02 |
| rs6726089 | 2 | 46418341 | C/G | *TMEM247* | intergenic | Novel | Novel | 2.40E-06 | 5.68E-03 |
| rs6853216 | 4 | 17969032 | T/A | *LCORL* | intronic | Novel | Confirmed | 1.50E-09 | 2.60E-06 |
| rs6918981 | 6 | 34270737 | A/T | *RP11-513I15.6* | intergenic | Novel | Novel | 1.10E-05 | 5.93E-03 |
| rs6948511 | 7 | 27939096 | C/G | *JAZF1* | intronic | Novel | Novel | 9.50E-05 | 2.15E-02 |
| rs7018178 | 8 | 141229111 | C/G | *SLC45A4* | intergenic | Novel | Confirmed | 1.40E-05 | 3.96E-03 |
| rs7196634 | 16 | 27202903 | T/A | *KDM8* | intergenic | Novel | Novel | 2.30E-05 | 2.32E-02 |
| rs7302663 | 12 | 93811355 | A/T | *CRADD* | intronic | Novel | Novel | 8.20E-05 | 2.50E-02 |
| rs730439 | 15 | 85391300 | C/G | *AKAP13* | intronic | Novel | Novel | 1.90E-04 | 2.82E-02 |
| rs7309412 | 12 | 102679315 | A/T | *LINC00485* | intergenic | Novel | Novel | 1.60E-06 | 4.52E-03 |
| rs732563 | 8 | 23488013 | C/G | *CTC-756D1.2* | intergenic | Novel | Novel | 5.70E-07 | 1.01E-03 |
| rs740672 | 4 | 17780641 | C/G | *FAM184B* | intronic | Novel | Novel | 2.60E-07 | 3.47E-04 |
| rs7429010 | 3 | 184437620 | G/C | *EIF2B5* | intergenic | Novel | Novel | 5.20E-05 | 4.48E-02 |
| rs7544210 | 1 | 22128649 | A/T | *WNT4* | intronic | Novel | Confirmed | 1.50E-05 | 5.57E-03 |
| rs7547731 | 1 | 21833655 | T/A | *HSPG2* | intronic | Novel | Novel | 6.80E-05 | 8.78E-03 |
| rs7549007 | 1 | 152083892 | C/G | *TCHHL1* | intergenic | Novel | Novel | 3.60E-05 | 8.16E-03 |
| rs757558 | 17 | 65565474 | T/A | *CTD-2535L24.2* | intergenic | Novel | Novel | 1.20E-05 | 1.83E-02 |
| rs7597315 | 2 | 46260221 | G/C | *EPAS1* | intergenic | Novel | Confirmed | 7.90E-11 | 8.50E-07 |
| rs7698621 | 4 | 95507414 | G/C | *UNC5C* | intronic | Novel | Novel | 1.40E-04 | 3.59E-02 |
| rs7846135 | 8 | 125489286 | A/T | *RP11-136O12.2* | intergenic | Novel | Novel | 2.80E-07 | 7.20E-04 |
| rs7965495 | 12 | 66037910 | A/T | *RP11-366L20.4* | intergenic | Novel | Novel | 1.10E-04 | 4.73E-02 |
| rs7974729 | 12 | 93818314 | T/A | *CRADD* | intronic | Novel | Novel | 4.40E-05 | 2.09E-02 |
| rs8008029 | 14 | 52979566 | T/A | *FERMT2* | intergenic | Novel | Novel | 8.20E-05 | 2.81E-02 |
| rs8039305 | 15 | 90879313 | C/G | *FURIN* | intronic | Novel | Novel | 4.90E-08 | 1.13E-06 |
| rs8056429 | 16 | 57078953 | A/T | *NLRC5* | intronic | Novel | Novel | 7.10E-06 | 1.02E-02 |
| rs8105944 | 19 | 51047598 | T/A | *KLK13* | intergenic | Novel | Novel | 2.70E-03 | 3.81E-02 |
| rs8108865 | 19 | 17761260 | T/A | *FCHO1* | intronic | Novel | Novel | 1.10E-03 | 3.11E-02 |
| rs8125378 | 20 | 31852354 | A/T | *DUSP15* | intergenic | Novel | Novel | 4.10E-05 | 6.64E-03 |
| rs817362 | 20 | 63928611 | A/T | *DNAJC5* | intronic | Novel | Novel | 1.50E-05 | 7.92E-03 |
| rs8182579 | 19 | 33418945 | T/A | *PEPD* | intronic | Novel | Confirmed | 1.80E-05 | 6.53E-03 |
| rs821551 | 1 | 155718789 | A/T | *DAP3* | intronic | Novel | Novel | 1.90E-05 | 6.67E-04 |
| rs851983 | 6 | 151703280 | G/C | *ESR1* | intronic | Novel | Confirmed | 1.50E-07 | 2.73E-04 |
| rs866919 | 10 | 30224354 | T/A | *RP11-305E6.1* | intergenic | Novel | Novel | 6.30E-04 | 9.87E-03 |
| rs889203 | 16 | 20039336 | T/A | *GPR139* | intronic | Novel | Confirmed | 1.30E-06 | 9.02E-04 |
| rs895964 | 12 | 26705133 | A/T | *ITPR2* | intronic | Novel | Confirmed | 1.20E-07 | 4.61E-04 |
| rs9303285 | 17 | 40344662 | C/G | *RARA* | intronic | Novel | Novel | 8.90E-05 | 3.39E-02 |
| rs9457107 | 6 | 165600735 | T/A | *PDE10A* | intronic | Novel | Novel | 3.10E-04 | 4.58E-02 |
| rs950805 | 1 | 39239786 | C/G | *MACF1* | intronic | Novel | Novel | 1.20E-04 | 4.02E-02 |
| rs9568036 | 13 | 48397800 | A/T | *LPAR6* | intronic | Novel | Novel | 5.90E-05 | 3.91E-02 |
| rs9611018 | 22 | 38759868 | T/A | *SUN2* | intergenic | Novel | Novel | 5.90E-05 | 4.78E-02 |
| rs965098 | 21 | 15185306 | A/T | *AF127577.12* | intergenic | Novel | Novel | 1.50E-03 | 2.35E-02 |
| rs9721852 | 9 | 89748077 | C/G | *RP5-1050E16.2* | intergenic | Novel | Novel | 1.10E-05 | 8.97E-03 |
| rs9877642 | 3 | 157069139 | A/T | *RP11-6F2.4* | intergenic | Novel | Novel | 2.20E-05 | 1.24E-02 |
| rs9903979 | 17 | 67761302 | C/G | *NOL11* | intergenic | Novel | Novel | 7.50E-05 | 2.12E-02 |
| rs9938631 | 16 | 67397901 | T/A | *ZDHHC1* | intronic | Novel | Novel | 2.50E-05 | 2.20E-02 |
| rs9962540 | 18 | 23138854 | A/T | *CABLES1* | intronic | Novel | Novel | 7.20E-06 | 1.26E-02 |
