# Supplementary material for: Identification of novel SNPs associated with coronary artery disease and birth weight using a pleiotropic cFDR method
Source: Aging (Albany NY). 2020 Dec 19;13(3):3618–44. doi: 10.18632/aging.202322 (PMC7906162; doi:10.18632/aging.202322)
Supplement: Supplementary Table 5 [file aging-13-202322-s006.pdf]

SUPPLEMENTARY TABLE

Supplementary Table 5. 19 SNPs in high LD (R^2> 0.6) with BW-associated loci.

SNP	Traits	Proxy SNP	R^2	*P*_value
rs10786156	Birth weight	rs2274224	0.996	8.00E-13
rs10840346	Birth weight	rs4444073	0.829	3.00E-15
rs1218565	Birth weight	rs6426985	0.832	7.00E-11
rs1319046	Birth weight	rs4965425	0.819	4.00E-09
rs1319859	Birth weight	rs11630479	0.849	9.00E-07
rs1415181	Birth weight	rs1244983	0.733	5.00E-10
rs1983127	Birth weight	rs9645500	0.732	1.00E-12
rs2423512	Birth weight	rs6040076	0.799	7.00E-09
rs2823025	Birth weight	rs2229742	0.814	2.00E-08
rs4812493	Birth weight	rs753381	0.720	3.00E-09
rs5765273	Birth weight	rs11704481	0.662	1.00E-08
rs6007030	Birth weight	rs11704481	0.603	1.00E-08
rs6072263	Birth weight	rs753381	0.724	3.00E-09
rs6918981	Birth length	rs1759645	0.663	7.00E-10
rs7309412	Birth weight	rs2647873	0.877	3.00E-12
rs7846135	Birth weight	rs8180991	0.828	1.00E-08
rs821551	Birth weight	rs670523	0.645	8.00E-12
rs889203	Birth weight	rs2045457	0.779	6.00E-09
rs895964	Birth weight	rs2306547	0.625	4.00E-13
