# Supplementary material for: Identification of novel SNPs associated with coronary artery disease and birth weight using a pleiotropic cFDR method
Source: Aging (Albany NY). 2020 Dec 19;13(3):3618–44. doi: 10.18632/aging.202322 (PMC7906162; doi:10.18632/aging.202322)
Supplement: Supplementary Table 6 [file aging-13-202322-s007.docx]

**Supplementary Table 6.** Conditional FDR values of 229 SNPs for BW given the CAD in validation dataset (cFDR ≤ 0.05).

| **SNP** | **Chr** | **Pos** | **Alt** | **Gene** | **Annotation** | ***P*_value** | **cFDR** | **Validation** |
| --- | --- | --- | --- | --- | --- | --- | --- | --- |
| rs10008032 | 4 | 38743861 | C/G | *RN5S158* | intergenic | 3.70E-05 | 1.76E-02 | Yes |
| rs1003573 | 7 | 44223857 | C/G | *CAMK2B* | intronic | 1.60E-05 | 1.90E-02 | Yes |
| rs10049090 | 3 | 157079913 | A/T | *RP11-6F2.4* | intergenic | 2.80E-42 | 2.08E-37 | Yes |
| rs1012626 | 6 | 20577330 | T/A | *CDKAL1* | intronic | 4.50E-15 | 1.20E-11 | Yes |
| rs1012635 | 6 | 20675064 | G/C | *CDKAL1* | intronic | 4.00E-10 | 2.35E-06 | Yes |
| rs10130093 | 14 | 89332778 | A/T | *FOXN3* | intronic | 3.30E-06 | 6.59E-03 | Yes |
| rs10200680 | 2 | 223097159 | C/T | *KCNE4* | intergenic | 2.70E-04 | 4.15E-02 | No |
| rs10221235 | 17 | 70097771 | C/G | *KCNJ16* | intronic | 4.10E-05 | 3.59E-02 | Yes |
| rs10283100 | 8 | 119583783 | G/C | *ENPP2* | missense | 1.80E-05 | 2.06E-02 | Yes |
| rs1042725 | 12 | 65964567 | T/A | *HMGA2* | 3'-UTR | 7.10E-32 | 7.21E-29 | Yes |
| rs1044299 | 1 | 176842737 | T/A | *PAPPA2* | 3'-UTR | 3.60E-06 | 4.25E-03 | Yes |
| rs10457487 | 6 | 127198089 | A/T | *RP11-73O6.4* | intergenic | 3.90E-05 | 2.08E-02 | Yes |
| rs10461018 | 3 | 46953752 | T/A | *CCDC12* | intronic | 2.70E-05 | 3.16E-02 | Yes |
| rs10494967 | 1 | 213828251 | A/T | *PROX1-AS1* | intronic | 5.40E-05 | 3.08E-02 | Yes |
| rs10514019 | 18 | 71669759 | C/G | *RP11-723G8.2* | intergenic | 1.40E-05 | 1.84E-02 | Yes |
| rs10743428 | 12 | 21952634 | T/C | *ABCC9* | intergenic | 2.40E-04 | 4.48E-02 | No |
| rs10753804 | 1 | 170347267 | C/T | *AL354732.1* | intergenic | 2.70E-04 | 3.18E-02 | No |
| rs10774625 | 12 | 111472415 | G/C | *ATXN2* | intronic | 3.40E-06 | 2.04E-05 | Yes |
| rs10786156 | 10 | 94254865 | G/C | *PLCE1* | intronic | 1.80E-07 | 1.43E-04 | Yes |
| rs10786706 | 10 | 102740902 | T/A | *SFXN2* | intergenic | 1.10E-03 | 3.90E-02 | Yes |
| rs10804733 | 3 | 148913142 | G/C | *RP11-680B3.2* | intergenic | 8.70E-05 | 2.68E-02 | Yes |
| rs10840346 | 11 | 10041452 | A/T | *SBF2* | intronic | 7.00E-07 | 3.56E-04 | Yes |
| rs10878353 | 12 | 65988752 | C/G | *HMGA2* | intergenic | 1.40E-08 | 3.59E-05 | Yes |
| rs10960954 | 9 | 13120381 | C/T | *MPDZ* | intronic | 4.00E-04 | 4.12E-02 | No |
| rs11066301 | 12 | 112433568 | G/C | *PTPN11* | intronic | 7.80E-04 | 9.88E-03 | Yes |
| rs11079803 | 17 | 47942535 | A/T | *PNPO* | intronic | 9.70E-06 | 7.22E-03 | Yes |
| rs11090046 | 22 | 41373079 | C/G | *TEF* | intronic | 4.70E-06 | 4.22E-03 | Yes |
| rs11125079 | 2 | 46505076 | T/A | *ATP6V1E2* | intergenic | 9.50E-06 | 1.12E-02 | Yes |
| rs11175992 | 12 | 65997616 | A/T | *RP11-366L20.4* | intergenic | 5.40E-06 | 7.40E-03 | Yes |
| rs11187076 | 10 | 92577206 | A/T | *IDE* | intergenic | 8.10E-08 | 1.54E-04 | Yes |
| rs11206803 | 1 | 56411837 | T/A | *RP4-710M16.2* | intergenic | 3.00E-03 | 4.44E-02 | Yes |
| rs1147322 | 9 | 122913322 | G/C | *ZBTB6* | 5'-UTR | 9.90E-11 | 7.30E-07 | Yes |
| rs11690295 | 2 | 111893739 | C/G | *MERTK* | intergenic | 1.90E-06 | 2.79E-03 | Yes |
| rs11853441 | 15 | 90856978 | G/C | *Metazoa_SRP* | intergenic | 1.70E-04 | 5.99E-03 | Yes |
| rs11889485 | 2 | 46257398 | A/T | *EPAS1* | intergenic | 2.00E-13 | 2.81E-09 | Yes |
| rs11961715 | 6 | 34127919 | G/A | *GRM4* | intronic | 2.10E-04 | 4.84E-02 | No |
| rs1202427 | 7 | 149240045 | A/T | *ZNF212* | intronic | 4.00E-06 | 2.15E-03 | Yes |
| rs1206028 | 19 | 42351015 | C/G | *MEGF8* | intronic | 3.90E-05 | 3.47E-02 | Yes |
| rs12148530 | 15 | 96542056 | C/G | *7SK* | intergenic | 6.30E-04 | 4.34E-02 | Yes |
| rs1218565 | 1 | 154845211 | T/A | *KCNN3* | intronic | 2.90E-06 | 5.22E-03 | Yes |
| rs12269934 | 11 | 58386843 | C/G | *OR5B3* | intergenic | 2.20E-05 | 2.23E-02 | Yes |
| rs12270978 | 11 | 2201585 | C/T | *MIR4686* | intergenic | 2.90E-04 | 4.23E-02 | No |
| rs12306172 | 12 | 54145221 | A/T | *RP11-834C11.11* | intergenic | 3.80E-06 | 1.52E-04 | Yes |
| rs12359055 | 10 | 24790442 | T/A | *PRTFDC1* | intergenic | 3.00E-04 | 3.13E-02 | Yes |
| rs12371967 | 12 | 65952934 | C/G | *HMGA2* | intronic | 2.20E-10 | 4.68E-07 | Yes |
| rs12517677 | 5 | 57736959 | G/A | *RP11* | intergenic | 1.20E-04 | 4.69E-02 | No |
| rs12536475 | 7 | 35284979 | A/T | *AC009531.2* | intergenic | 2.20E-05 | 6.96E-03 | Yes |
| rs12623454 | 2 | 120568721 | C/G | *AC073257.1* | intergenic | 2.40E-05 | 5.43E-03 | Yes |
| rs12643660 | 4 | 105465603 | G/C | *PPA2* | intronic | 3.50E-05 | 2.82E-02 | Yes |
| rs12656216 | 5 | 36160566 | A/T | *SKP2* | intronic | 2.00E-06 | 2.74E-03 | Yes |
| rs12722772 | 1 | 176763612 | T/C | *PAPPA2* | intronic | 2.60E-04 | 4.84E-02 | No |
| rs12828089 | 12 | 46210774 | A/T | *SLC38A1* | intronic | 3.70E-05 | 3.69E-02 | Yes |
| rs12865243 | 13 | 40104683 | A/T | *LINC00332* | intergenic | 3.00E-06 | 7.44E-03 | Yes |
| rs1293935 | 6 | 151696823 | C/T | *ESR1* | intronic | 2.20E-10 | 4.05E-07 | No |
| rs13035774 | 2 | 24135782 | T/A | *AC008073.6* | intronic | 2.40E-04 | 2.57E-02 | Yes |
| rs1319046 | 15 | 98626817 | T/A | *RP11-35O15.1* | intergenic | 6.40E-06 | 4.34E-03 | Yes |
| rs1319859 | 15 | 98687034 | A/T | *IGF1R* | intronic | 5.00E-04 | 4.11E-02 | Yes |
| rs1319869 | 15 | 98669256 | T/A | *IGF1R* | intronic | 7.40E-06 | 4.72E-03 | Yes |
| rs1319888 | 17 | 49469749 | C/G | *RP11-81K2.1* | intergenic | 3.00E-05 | 2.70E-02 | Yes |
| rs1361024 | 6 | 151749793 | A/T | *ESR1* | intronic | 8.00E-06 | 1.46E-02 | Yes |
| rs1384539 | 3 | 157094152 | A/T | *RP11-6F2.4* | intronic | 2.30E-05 | 2.39E-02 | Yes |
| rs1389923 | 6 | 165747283 | T/A | *RP11-252P19.3* | intergenic | 1.50E-05 | 9.77E-03 | Yes |
| rs1415181 | 1 | 214857808 | C/G | *KCNK2* | intergenic | 3.70E-06 | 8.62E-03 | Yes |
| rs1415701 | 6 | 130024690 | A/T | *L3MBTL3* | intronic | 4.00E-11 | 2.91E-07 | Yes |
| rs1425661 | 4 | 67193004 | C/G | *RP11-584P21.2* | intergenic | 2.80E-05 | 1.87E-02 | Yes |
| rs143384 | 20 | 35437976 | G/C | *GDF5* | 5'-UTR | 6.40E-07 | 1.67E-03 | Yes |
| rs1451156 | 2 | 46605463 | T/A | *PIGF* | intronic | 2.60E-05 | 3.12E-02 | Yes |
| rs1475643 | 10 | 102786710 | T/A | *WBP1L* | intronic | 1.50E-04 | 3.48E-02 | Yes |
| rs1488691 | 1 | 91867308 | G/C | *TGFBR3* | intronic | 1.40E-05 | 7.21E-03 | Yes |
| rs149514 | 5 | 65555910 | A/T | *CENPK* | intronic | 8.30E-05 | 1.18E-02 | Yes |
| rs1547669 | 6 | 33807864 | G/C | *MLN* | intergenic | 1.00E-05 | 1.41E-02 | Yes |
| rs1548304 | 22 | 42295232 | T/A | *TCF20* | intergenic | 6.90E-06 | 9.63E-03 | Yes |
| rs1566383 | 3 | 2304089 | T/C | *CNTN4* | intronic | 1.30E-04 | 4.61E-02 | No |
| rs1580278 | 4 | 103219691 | A/T | *CENPE* | intergenic | 3.10E-05 | 3.29E-02 | Yes |
| rs1638410 | 10 | 116766015 | C/G | *HSPA12A* | intergenic | 5.70E-05 | 9.93E-03 | Yes |
| rs16887484 | 5 | 57859481 | G/C | *AC116606.1* | intergenic | 3.20E-05 | 8.70E-03 | Yes |
| rs16952999 | 18 | 936560 | T/A | *RP11-672L10.1* | intergenic | 4.30E-05 | 1.12E-02 | Yes |
| rs17015692 | 4 | 89502952 | T/A | *RP11-115D19.1* | intergenic | 2.80E-05 | 1.91E-02 | Yes |
| rs17058570 | 9 | 73167639 | C/T | *ANXA1* | intronic | 3.60E-05 | 3.00E-02 | No |
| rs17111909 | 1 | 55320101 | A/G | *7SK* | intergenic | 8.20E-05 | 4.27E-02 | No |
| rs17133917 | 7 | 50641953 | G/A | *GRB10* | intronic | 7.40E-05 | 4.07E-02 | No |
| rs17290714 | 3 | 47854215 | T/A | *MAP4* | intronic | 1.80E-05 | 2.09E-02 | Yes |
| rs17384555 | 18 | 5618174 | T/A | *EPB41L3* | intergenic | 2.30E-05 | 1.66E-02 | Yes |
| rs17472967 | 4 | 78704724 | A/T | *RP11-792D21.2* | intergenic | 1.10E-04 | 4.83E-02 | Yes |
| rs17566087 | 10 | 69219200 | G/C | *RP11-227H15.4* | intergenic | 2.30E-05 | 6.16E-03 | Yes |
| rs17745230 | 2 | 46263191 | T/A | *EPAS1* | intergenic | 9.10E-07 | 1.43E-03 | Yes |
| rs17767418 | 17 | 30479276 | T/A | *GOSR1* | intronic | 2.60E-08 | 3.26E-05 | Yes |
| rs17826255 | 17 | 31006498 | C/G | *RNF135* | intronic | 2.80E-05 | 2.59E-02 | Yes |
| rs1797081 | 10 | 16832566 | C/G | *CUBN* | intronic | 1.50E-05 | 2.16E-02 | Yes |
| rs1983127 | 10 | 69230145 | T/A | *RP11-227H15.4* | intronic | 3.70E-05 | 3.76E-02 | Yes |
| rs2013116 | 12 | 92502603 | T/A | *RP11-693J15.4* | intergenic | 9.70E-05 | 1.90E-02 | Yes |
| rs2087826 | 4 | 144718166 | A/T | *HHIP* | intronic | 4.00E-09 | 1.66E-05 | Yes |
| rs2150052 | 9 | 111182787 | T/A | *RP11-202G18.1* | intergenic | 2.80E-08 | 6.75E-05 | Yes |
| rs2160875 | 12 | 4418156 | T/A | *FGF6* | intergenic | 1.30E-04 | 2.69E-02 | Yes |
| rs2186797 | 11 | 70161664 | T/C | *ANO1* | missense | 2.70E-05 | 1.83E-02 | No |
| rs2191883 | 7 | 35233679 | C/G | *TBX20* | intronic | 4.20E-06 | 1.95E-03 | Yes |
| rs2206734 | 6 | 20694653 | C/T | *CDKAL1* | intronic | 2.40E-17 | 3.92E-13 | No |
| rs2214409 | 4 | 105111788 | T/A | *RP11-556I14.1* | intergenic | 6.00E-06 | 1.21E-02 | Yes |
| rs222837 | 17 | 7229237 | T/A | *DVL2* | synonymous | 3.90E-07 | 1.45E-03 | Yes |
| rs2243621 | 6 | 31464043 | C/T | *HCP5* | 3'-UTR | 2.40E-03 | 4.50E-02 | No |
| rs2287859 | 19 | 17774461 | A/G | *FCHO1* | synonymous | 5.10E-04 | 4.82E-02 | No |
| rs2288291 | 11 | 12474060 | C/G | *PARVA* | intronic | 4.60E-05 | 2.28E-02 | Yes |
| rs2290228 | 7 | 128748594 | A/T | *CALU* | missense | 1.90E-04 | 3.98E-02 | Yes |
| rs2296742 | 6 | 33692016 | A/T | *ITPR3* | intronic | 1.40E-04 | 2.52E-02 | Yes |
| rs2298229 | 13 | 53028835 | G/C | *OLFM4* | 5'-UTR | 4.00E-05 | 3.93E-02 | Yes |
| rs2306531 | 3 | 157099873 | T/A | *RP11-6F2.4* | intronic | 8.60E-06 | 1.43E-02 | Yes |
| rs2339940 | 2 | 24028917 | T/A | *MFSD2B* | intergenic | 4.50E-07 | 1.16E-04 | Yes |
| rs2423512 | 20 | 10705728 | C/G | *JAG1* | intergenic | 2.10E-07 | 2.38E-04 | Yes |
| rs2426778 | 20 | 58718421 | G/A | *NPEPL1* | missense | 9.70E-05 | 3.79E-02 | No |
| rs2488071 | 10 | 92739820 | A/G | *Y_RNA* | intergenic | 2.70E-08 | 2.48E-05 | No |
| rs2493995 | 6 | 20753888 | A/T | *CDKAL1* | intronic | 2.80E-05 | 2.80E-02 | Yes |
| rs2505126 | 10 | 30100897 | A/T | *KIAA1462* | intergenic | 2.10E-04 | 2.76E-02 | Yes |
| rs2540074 | 9 | 123209217 | C/G | *STRBP* | intronic | 1.90E-10 | 1.11E-06 | Yes |
| rs268691 | 19 | 40443765 | C/G | *SERTAD3* | intronic | 4.20E-05 | 2.88E-02 | Yes |
| rs2709370 | 2 | 207517878 | A/G | *AC007879.5* | intergenic | 7.50E-05 | 4.44E-02 | No |
| rs2715878 | 2 | 9375405 | G/C | *ASAP2* | intronic | 4.70E-05 | 2.48E-02 | Yes |
| rs2782980 | 10 | 114021768 | C/G | *ADRB1* | intergenic | 2.50E-05 | 1.21E-02 | Yes |
| rs2807376 | 1 | 22186354 | C/T | *WNT4* | intergenic | 1.50E-04 | 4.16E-02 | No |
| rs2823025 | 21 | 15052100 | G/C | *AF127577.1* | 3'-UTR | 7.90E-07 | 1.03E-03 | Yes |
| rs28536742 | 9 | 95520811 | T/A | *PTCH1* | intergenic | 2.10E-11 | 1.96E-07 | Yes |
| rs2886070 | 1 | 156004180 | A/T | *RP11-336K24.4* | intergenic | 3.50E-08 | 7.70E-05 | Yes |
| rs293339 | 6 | 130402339 | G/A | *TMEM200A* | intronic | 2.80E-05 | 1.87E-02 | No |
| rs3198697 | 16 | 15036083 | T/A | *PDXDC1* | synonymous | 3.50E-05 | 7.21E-03 | Yes |
| rs328294 | 8 | 38503861 | C/T | *C8orf86* | intergenic | 1.20E-04 | 4.83E-02 | No |
| rs328890 | 7 | 34973837 | A/T | *DPY19L1* | intronic | 2.10E-04 | 3.84E-02 | Yes |
| rs32902 | 5 | 77013087 | G/C | *AGGF1* | intergenic | 2.80E-05 | 1.85E-02 | Yes |
| rs333550 | 15 | 65965070 | T/C | *MEGF11* | missense | 1.10E-04 | 2.18E-02 | No |
| rs33848 | 19 | 33533344 | G/A | *PEPD* | intergenic | 1.40E-04 | 4.92E-02 | No |
| rs3756668 | 5 | 68300260 | A/T | *PIK3R1* | 3'-UTR | 1.70E-05 | 2.11E-03 | Yes |
| rs3772587 | 3 | 148859129 | T/A | *CPB1* | intronic | 1.50E-06 | 1.93E-03 | Yes |
| rs3795521 | 1 | 214641228 | T/A | *CENPF* | synonymous | 3.00E-06 | 3.83E-03 | Yes |
| rs3849774 | 5 | 39437520 | G/C | *DAB2* | intergenic | 5.80E-05 | 4.90E-02 | Yes |
| rs3907223 | 5 | 16381408 | C/T | *RP1* | intergenic | 2.40E-04 | 4.00E-02 | No |
| rs4130707 | 5 | 85071655 | G/A | *CTC-384G19.1* | intergenic | 9.50E-05 | 2.92E-02 | No |
| rs4143341 | 7 | 159262263 | G/C | *VIPR2* | intergenic | 3.20E-06 | 4.65E-03 | Yes |
| rs4144650 | 6 | 20938690 | A/T | *CDKAL1* | intronic | 6.70E-08 | 1.27E-04 | Yes |
| rs4233701 | 2 | 23706216 | C/G | *KLHL29* | intronic | 5.20E-07 | 1.15E-04 | Yes |
| rs4428060 | 2 | 46263662 | T/A | *EPAS1* | intergenic | 1.70E-09 | 4.93E-06 | Yes |
| rs4430500 | 11 | 10232824 | T/C | *SBF2* | intronic | 6.60E-06 | 1.13E-02 | No |
| rs455567 | 6 | 33284338 | G/A | *WDR46* | intronic | 2.00E-04 | 2.71E-02 | No |
| rs4677887 | 3 | 123381376 | G/C | *ADCY5* | intronic | 2.30E-12 | 3.94E-08 | Yes |
| rs4699908 | 5 | 57618434 | A/T | *CTD-2023N9.3* | intergenic | 4.50E-06 | 9.56E-03 | Yes |
| rs4704942 | 5 | 158466352 | C/G | *RP11-542A14.1* | intergenic | 6.10E-08 | 1.83E-05 | Yes |
| rs4710945 | 6 | 20744843 | T/A | *CDKAL1* | intronic | 9.20E-10 | 2.62E-06 | Yes |
| rs4712542 | 6 | 20772587 | T/A | *RP3-348I23.2* | intronic | 2.90E-05 | 2.18E-02 | Yes |
| rs4753073 | 11 | 92984309 | G/A | *MTNR1B* | intergenic | 2.90E-04 | 4.97E-02 | No |
| rs475931 | 11 | 104914727 | G/C | *CASP12* | intronic | 3.60E-05 | 3.65E-02 | Yes |
| rs4762119 | 12 | 65770055 | T/A | *HMGA2* | intronic | 1.10E-05 | 4.60E-03 | Yes |
| rs4793636 | 17 | 50062136 | A/T | *ITGA3* | intronic | 4.10E-05 | 3.98E-02 | Yes |
| rs4812493 | 20 | 41320745 | T/A | *ZHX3* | intergenic | 8.20E-06 | 2.15E-03 | Yes |
| rs4823047 | 22 | 29553889 | C/T | *THOC5* | intergenic | 8.30E-05 | 3.55E-02 | No |
| rs4833051 | 4 | 38461997 | A/G | *RP11* | intergenic | 3.20E-04 | 4.49E-02 | No |
| rs4853831 | 2 | 1809892 | C/G | *MYT1L* | intronic | 5.10E-05 | 4.20E-02 | Yes |
| rs4875812 | 8 | 1811300 | G/C | *MIR596* | intergenic | 4.60E-05 | 7.71E-03 | Yes |
| rs502467 | 3 | 172009573 | C/G | *FNDC3B* | intergenic | 1.40E-03 | 4.00E-02 | Yes |
| rs513349 | 6 | 33573942 | A/G | *BAK1* | intronic | 3.30E-04 | 4.44E-02 | No |
| rs5742915 | 15 | 74044292 | C/G | *PML* | missense | 3.10E-05 | 3.50E-03 | Yes |
| rs5765273 | 22 | 45352071 | G/C | *SMC1B* | intronic | 9.30E-06 | 9.94E-03 | Yes |
| rs6007030 | 22 | 45434479 | G/C | *RP1-102D24.5* | intergenic | 2.50E-05 | 2.80E-02 | Yes |
| rs6016377 | 20 | 40544088 | T/A | *SNORD112* | intergenic | 3.60E-10 | 1.83E-07 | Yes |
| rs6057610 | 20 | 32653587 | C/G | *C20orf203* | intergenic | 3.00E-10 | 6.13E-07 | Yes |
| rs6062314 | 20 | 63778360 | C/T | *ZBTB46* | intronic | 2.40E-04 | 3.70E-02 | No |
| rs6072263 | 20 | 41076906 | C/G | *TOP1* | intronic | 1.10E-05 | 2.84E-03 | Yes |
| rs6075924 | 20 | 22531891 | C/G | *RP11-216C10.1* | intergenic | 4.80E-06 | 1.97E-03 | Yes |
| rs611003 | 11 | 69630516 | A/T | *CCND1* | intergenic | 7.10E-06 | 4.25E-03 | Yes |
| rs630014 | 9 | 133274306 | G/C | *ABO* | intronic | 4.10E-04 | 9.57E-03 | Yes |
| rs6424243 | 1 | 232632402 | A/T | *U6* | intergenic | 1.10E-05 | 9.10E-03 | Yes |
| rs6437106 | 2 | 157553684 | T/A | *ACVR1C* | intronic | 2.70E-05 | 2.85E-02 | Yes |
| rs646596 | 1 | 209826803 | C/G | *DIEXF* | intergenic | 9.60E-05 | 2.57E-02 | Yes |
| rs6484487 | 11 | 30511445 | C/G | *MPPED2* | intronic | 1.60E-05 | 5.11E-03 | Yes |
| rs6511689 | 19 | 10210413 | C/G | *S1PR2* | intergenic | 2.90E-05 | 7.70E-03 | Yes |
| rs663344 | 9 | 95554049 | C/G | *PTCH1* | intergenic | 2.90E-05 | 2.14E-02 | Yes |
| rs6673081 | 1 | 155017119 | C/G | *ZBTB7B* | 3'-UTR | 5.30E-10 | 2.11E-07 | Yes |
| rs6700896 | 1 | 65624099 | T/A | *LEPR* | intronic | 3.60E-05 | 2.49E-02 | Yes |
| rs670950 | 19 | 43777410 | C/G | *KCNN4* | intronic | 2.30E-04 | 2.69E-02 | Yes |
| rs6713510 | 2 | 226169783 | A/T | *AC068138.1* | intronic | 6.70E-04 | 1.63E-02 | Yes |
| rs6726089 | 2 | 46418341 | C/G | *TMEM247* | intergenic | 2.40E-06 | 6.55E-03 | Yes |
| rs6845 | 20 | 63778360 | C/T | *EIF2B5* | 3'-UTR | 9.60E-05 | 4.78E-02 | No |
| rs6853216 | 4 | 17969032 | T/A | *LCORL* | intronic | 1.50E-09 | 1.42E-06 | Yes |
| rs6908425 | 6 | 20728500 | T/C | *CDKAL1* | intronic | 9.80E-05 | 4.55E-02 | No |
| rs6918981 | 6 | 34270737 | A/T | *RP11-513I15.6* | intergenic | 1.10E-05 | 1.63E-03 | Yes |
| rs6948511 | 7 | 27939096 | C/G | *JAZF1* | intronic | 9.50E-05 | 2.99E-02 | Yes |
| rs6994725 | 8 | 65932508 | G/A | *DNAJC5B* | intergenic | 2.80E-04 | 4.81E-02 | No |
| rs7018178 | 8 | 141229111 | C/G | *SLC45A4* | intergenic | 1.40E-05 | 4.89E-03 | Yes |
| rs7196634 | 16 | 27202903 | T/A | *KDM8* | intergenic | 2.30E-05 | 2.88E-02 | Yes |
| rs7302663 | 12 | 93811355 | A/T | *CRADD* | intronic | 8.20E-05 | 1.75E-02 | Yes |
| rs730439 | 15 | 85391300 | C/G | *AKAP13* | intronic | 1.90E-04 | 1.94E-02 | Yes |
| rs7309412 | 12 | 102679315 | A/T | *LINC00485* | intergenic | 1.60E-06 | 4.19E-03 | Yes |
| rs732563 | 8 | 23488013 | C/G | *CTC-756D1.2* | intergenic | 5.70E-07 | 1.42E-03 | Yes |
| rs734351 | 11 | 2134983 | G/A | *IGF2* | intronic | 1.90E-06 | 2.29E-03 | No |
| rs740672 | 4 | 17780641 | C/G | *FAM184B* | intronic | 2.60E-07 | 3.62E-04 | Yes |
| rs742086 | 22 | 42156247 | T/C | *TCF20* | intergenic | 5.80E-06 | 7.19E-03 | No |
| rs7429010 | 3 | 184437620 | G/C | *EIF2B5* | intergenic | 5.20E-05 | 3.86E-02 | Yes |
| rs7544210 | 1 | 22128649 | A/T | *WNT4* | intronic | 1.50E-05 | 3.21E-03 | Yes |
| rs7547731 | 1 | 21833655 | T/A | *HSPG2* | intronic | 6.80E-05 | 2.39E-02 | Yes |
| rs7549007 | 1 | 152083892 | C/G | *TCHHL1* | intergenic | 3.60E-05 | 2.27E-02 | Yes |
| rs757558 | 17 | 65565474 | T/A | *CTD-2535L24.2* | intergenic | 1.20E-05 | 1.45E-02 | Yes |
| rs7597315 | 2 | 46260221 | G/C | *EPAS1* | intergenic | 7.90E-11 | 9.50E-07 | Yes |
| rs7612543 | 3 | 141439370 | G/A | *ZBTB38* | intronic | 1.20E-04 | 4.82E-02 | No |
| rs7698621 | 4 | 95507414 | G/C | *UNC5C* | intronic | 1.40E-04 | 3.59E-02 | Yes |
| rs772313 | 13 | 98818805 | C/T | *DOCK9* | intronic | 1.50E-04 | 4.66E-02 | No |
| rs7768493 | 6 | 5594664 | C/T | *FARS2* | intronic | 1.90E-04 | 4.65E-02 | No |
| rs7846135 | 8 | 125489286 | A/T | *RP11-136O12.2* | intergenic | 2.80E-07 | 4.75E-04 | Yes |
| rs7965495 | 12 | 66037910 | A/T | *RP11-366L20.4* | intergenic | 1.10E-04 | 2.26E-02 | Yes |
| rs7974729 | 12 | 93818314 | T/A | *CRADD* | intronic | 4.40E-05 | 2.13E-02 | Yes |
| rs8039305 | 15 | 90879313 | C/G | *FURIN* | intronic | 4.90E-08 | 3.92E-07 | Yes |
| rs8056429 | 16 | 57078953 | A/T | *NLRC5* | intronic | 7.10E-06 | 9.57E-03 | Yes |
| rs8125378 | 20 | 31852354 | A/T | *DUSP15* | intergenic | 4.10E-05 | 4.51E-03 | Yes |
| rs817362 | 20 | 63928611 | A/T | *DNAJC5* | intronic | 1.50E-05 | 2.06E-03 | Yes |
| rs8182579 | 19 | 33418945 | T/A | *PEPD* | intronic | 1.80E-05 | 6.84E-03 | Yes |
| rs821551 | 1 | 155718789 | A/T | *DAP3* | intronic | 1.90E-05 | 4.53E-03 | Yes |
| rs833715 | 12 | 102674622 | A/G | *LINC00485* | intergenic | 2.50E-04 | 4.59E-02 | No |
| rs866919 | 10 | 30224354 | T/A | *RP11-305E6.1* | intergenic | 6.30E-04 | 2.45E-02 | Yes |
| rs889203 | 16 | 20039336 | T/A | *GPR139* | intronic | 1.30E-06 | 7.10E-04 | Yes |
| rs895964 | 12 | 26705133 | A/T | *ITPR2* | intronic | 1.20E-07 | 3.91E-04 | Yes |
| rs935172 | 2 | 26581379 | T/C | *CIB4* | missense | 9.30E-05 | 3.02E-02 | No |
| rs9457107 | 6 | 165600735 | T/A | *PDE10A* | intronic | 3.10E-04 | 3.41E-02 | Yes |
| rs9492469 | 6 | 130169033 | G/A | *SAMD3* | intronic | 1.80E-05 | 9.78E-03 | No |
| rs950805 | 1 | 39239786 | C/G | *MACF1* | intronic | 1.20E-04 | 2.34E-02 | Yes |
| rs9568036 | 13 | 48397800 | A/T | *LPAR6* | intronic | 5.90E-05 | 3.67E-02 | Yes |
| rs9611018 | 22 | 38759868 | T/A | *SUN2* | intergenic | 5.90E-05 | 4.70E-02 | Yes |
| rs965098 | 21 | 15185306 | A/T | *AF127577.12* | intergenic | 1.50E-03 | 4.08E-02 | Yes |
| rs972088 | 7 | 127682935 | A/G | *SND1* | intronic | 2.60E-04 | 4.93E-02 | No |
| rs9721852 | 9 | 89748077 | C/G | *RP5-1050E16.2* | intergenic | 1.10E-05 | 1.74E-02 | Yes |
| rs9877642 | 3 | 157069139 | A/T | *RP11-6F2.4* | intergenic | 2.20E-05 | 2.86E-02 | Yes |
| rs9903979 | 17 | 67761302 | C/G | *NOL11* | intergenic | 7.50E-05 | 2.98E-02 | Yes |
| rs9909792 | 17 | 76541492 | G/A | *PRCD* | intronic | 3.10E-04 | 4.41E-02 | No |
| rs9938631 | 16 | 67397901 | T/A | *ZDHHC1* | intronic | 2.50E-05 | 1.70E-02 | Yes |
| rs9962540 | 18 | 23138854 | A/T | *CABLES1* | intronic | 7.20E-06 | 7.16E-03 | Yes |
