# Supplementary material for: Identification of novel SNPs associated with coronary artery disease and birth weight using a pleiotropic cFDR method
Source: Aging (Albany NY). 2020 Dec 19;13(3):3618–44. doi: 10.18632/aging.202322 (PMC7906162; doi:10.18632/aging.202322)
Supplement: Supplementary Tables 7, 8, 9, 10, 11 and 12 [file aging-13-202322-s008.pdf]

SUPPLEMENTARY TABLES

Supplementary Table 7. MetaQTL effects of significant SNPs.

SNP	Traits	Metabolics	Related disease or biofunctions(PMID)	Sample Type	*P*_value	Source
rs10774625	Pleiotropic	5-hydroxytryptophan	Depression(31071306)	serum	6.52E-06	SI data (Long et al.)
		hypoxanthine	Coronary artery disease (18651524)	serum	1.29E-06	SI data (Long et al.)
		quinolinate	Acute kidney injury (31055583)	serum	3.21E-06	SI data (Long et al.)
		C-glycosyltryptophan		serum	5.41E-05	SI data (Shin et al.)
		gamma-glutamylleucine		serum	6.14E-05	SI data (Shin et al.)
		kynurenine	Blood pressure (20190767)	serum	1.47E-16	SI data (Shin et al.)
		erythronate	Cirrhosis (29291380)	serum	5.78E-05	SI data (Shin et al.)
rs11066301	Pleiotropic	kynurenine	Blood pressure (20190767)	serum	2.93E-11	SI data (Shin et al.)
		erythronate	Cirrhosis (29291380)	serum	1.71E-05	SI data (Shin et al.)
rs11172113	Pleiotropic	SM C18:1	Coronary artery disease (28431006)	serum	9.21E-05	SI data (Draisma et al.)
rs630014	Pleiotropic	glycylglycine	Alzheimer's disease (28951883)	serum	6.26E-06	SI data (Long et al.)
		ADpSGEGDFXAEGGGVR	T2D (30372032)	serum	3.53E-09	SI data (Shin et al.)
		ADpSGEGDFXAEGGGVR	T2D (30372032)	serum	1.64E-06	SI data (Suhre et al.)
rs10791643	CAD	propylene glycol	Hyperketonemia (27638258)	urine	4.60E-05	SI data (Raffler et al.)
rs11066301	CAD	kynurenine	Blood pressure (20190767)	serum	2.93E-11	SI data (Shin et al.)
		erythronate	Cirrhosis (29291380)	serum	1.71E-05	SI data (Shin et al.)
rs11668477	CAD	cholesterol	Multiple metabolic disease (28319895)	serum	7.69E-05	SI data (Shin et al.)
rs1418278	CAD	X-14057		serum	2.98E-05	SI data (Shin et al.)
rs1541853	CAD	PC aa C32:2		serum	9.24E-05	SI data (Draisma et al.)
rs3811417	CAD	nonanoylcarnitine		serum	9.50E-05	SI data (Shin et al.)
rs3918291	CAD	1,7-dimethylurate		serum	5.41E-05	SI data (Suhre et al.)
rs405509	CAD	X-11820		serum	4.82E-11	SI data (Shin et al.)
rs4245791	CAD	X-12063		serum	3.07E-08	SI data (Shin et al.)
rs445925	CAD	palmitoyl-linoleoyl-glycerol		serum	2.11E-06	SI data (Long et al.)
		17beta-diol monosulfate		serum	2.07E-06	SI data (Long et al.)
		oleoyl-linoleoyl-glycerol		serum	7.34E-06	SI data (Long et al.)
		Tetradecenoylcarnitine	Aging-related Diseases (30498825)	serum	3.00E-05	SI data (Draisma et al.)
		PC aa C28:1		serum	6.54E-06	SI data (Draisma et al.)
		palmitoyl sphingomyelin	Dyslipidemia (18299615)	serum	1.97E-09	SI data (Shin et al.)
		cholesterol	Multiple metabolic disease (28319895)	serum	4.10E-10	SI data (Shin et al.)
rs4895390	CAD	2-hydroxyacetaminophen sulfate	Children obesity (30253079)	serum	8.89E-05	SI data (Shin et al.)
rs624249	CAD	X-12798		serum	2.38E-11	SI data (Long et al.)
rs6922782	CAD	X-12411		serum	6.65E-06	SI data (Long et al.)
rs998584	CAD	SM C16:1		serum	2.76E-05	SI data (Draisma et al.)
rs10221235	BW	PC aa C38:6		serum	1.66E-05	SI data (Draisma et al.)
rs1042725	BW	serine	Multiple metabolic disease (28319895)	serum	7.98E-07	SI data (Shin et al.)
rs10786156	BW	cyclo(leu-pro)		serum	1.26E-05	SI data (Shin et al.)
rs10786706	BW	X-12212		serum	2.68E-05	SI data (Shin et al.)
rs11125079	BW	HWESASXX	Blood pressure (27129722)	serum	7.34E-05	SI data (Suhre et al.)
rs11187076	BW	pregnen-diol disulfate		serum	1.55E-07	SI data (Long et al.)
rs12371967	BW	malate		serum	2.47E-05	SI data (Shin et al.)
rs12656216	BW	lysine	birth weight (19067286)	serum	2.82E-05	SI data (Shin et al.)
rs1389923	BW	hydroxyphenylacetic acid monosulfate		serum	7.18E-06	SI data (Shin et al.)

rs16887484	BW	guanosine		serum	8.82E-07	SI data (Shin et al.)
		inosine		serum	4.51E-05	SI data (Shin et al.)
rs1797081	BW	X-12749		serum	5.74E-05	SI data (Shin et al.)
rs2087826	BW	cotinine		serum	3.39E-05	SI data (Shin et al.)
rs2497304	BW	21-hydroxypregnenolone disulfate		serum	3.37E-06	SI data (Long et al.)
		dehydroisoandrosterone sulfate		serum	8.55E-05	SI data (Shin et al.)
rs3198697	BW	1-dihomo-linolenoyl-GPC		serum	4.53E-06	SI data (Long et al.)
		dihomo-linolenate	Oxidative stress (24760997)	serum	1.56E-07	SI data (Shin et al.)
		1-eicosatrienoylglycerophosphocholine		serum	2.35E-05	SI data (Suhre et al.)
rs3849774	BW	1-(1-enyl-stearoyl)-2-docosahexaenoyl-GPE		serum	8.36E-06	SI data (Long et al.)
rs4428060	BW	ADpSGEGDFXAEGGGVR	T2D (30372032)	serum	5.56E-05	SI data (Shin et al.)
		ADpSGEGDFXAEGGGVR	T2D (30372032)	serum	6.92E-05	SI data (Suhre et al.)
rs4712542	BW	dehydroisoandrosterone sulfate		serum	4.09E-05	SI data (Shin et al.)
rs475931	BW	4-hydroxyhippurate		serum	1.82E-05	SI data (Shin et al.)
rs4812493	BW	ADSGEGDFXAEGGGVR	T2D (30372032)	serum	6.73E-05	SI data (Shin et al.)
rs4875812	BW	deoxycholate		serum	6.12E-05	SI data (Shin et al.)
rs533318	BW	tiglyl carnitine		serum	4.81E-05	SI data (Suhre et al.)
rs6948511	BW	X-11795		serum	3.30E-06	SI data (Shin et al.)
rs8108865	BW	HWESASXX	Blood pressure (27129722)	serum	7.05E-05	SI data (Suhre et al.)
rs889203	BW	3-methylxanthine		serum	9.95E-06	SI data (Suhre et al.)

Supplementary Table 8. 9 pleiotropic SNPs also associated with other phenotypes.

SNP	Chr	Pos	Alt	metaQTL/pQTL/meQTL/eQTL	ccFDR	Traits	PMID
rs10774625	12	111472415	A/T	metaQTL/pQTL/meQTL/eQTL(3 hits)	3.06E-05	Colorectal cancer	29547645
						Hashimoto's thyroiditis	27268232
						Systemic lupus	27906046
						Type 1 diabetes	24936253
						Hypertension	19430479
rs11066301	12	112433568	A/T	metaQTL/meQTL/eQTL(1 hit)	6.50E-03	Hematological parameters	19820697
rs11172113	12	57133500	T/A	metaQTL/meQTL/eQTL(4 hits)	3.18E-02	Headache	29397368
						Migraine	27322543
						Pulmonary function	21946350
rs3756668	5	68300260	G/C		1.32E-02	Type 2 diabetes	29893513
						Small cell lung cancer	28280736
						Endometrial cancer	22146979
rs630014	9	133274306	A/T	metaQTL/meQTL/eQTL(9 hits)	1.16E-02	Pancreatic ductal adenocarcinoma	23816557
						Venous thromboembolism	21463476
						Pancreatic cancer	22523087
rs670950	19	43777410	T/A	eQTL(1 hit)	3.15E-02	Vascular diseases	19644414
rs6713510	2	226169783	G/C		1.29E-02	Fasting plasma glucose	21188353
rs8039305	15	90879313	T/A	meQTL/eQTL(27 hits)	3.77E-06	Hypertension	28686695
rs821551	1	155718789	C/G	meQTL/eQTL(50 hits)	1.08E-02	Osteoporotic fractures	21760914

Supplementary Table 9. Conjunction cFDR for 17 pleiotropic SNPs in CAD and BW in validation dataset (ccFDR ≤ 0.05).

SNP	Chr	Pos	Alt	Gene	Annotation	cFDR.CAD	cFDR.BW	ccFDR	Validation
rs1042725	12	65964567	C/T	*HMGA2*	3'-UTR	1.05E-02	7.21E-29	1.05E-02	No
rs10774625	12	111472415	A/T	*ATXN2*	intronic	5.16E-12	2.04E-05	2.04E-05	Yes
rs11066301	12	112433568	A/T	*PTPN11*	intronic	9.98E-05	9.88E-03	9.88E-03	Yes
rs11206803	1	56411837	C/G	*AC119674.2*	intronic	4.86E-03	4.44E-02	4.44E-02	Yes
rs11853441	15	90856978	T/G	*Metazoa_SRP*	intergenic	9.38E-03	5.99E-03	9.38E-03	No
rs12306172	12	54145221	G/C	*SMUG1*	intronic	2.20E-03	1.52E-04	2.20E-03	Yes
rs2243621	6	31464043	C/T	*HCP5*	3'-UTR	2.56E-02	4.50E-02	4.50E-02	No
rs2339940	2	24028917	G/C	*MFSD2B*	intronic	4.45E-02	1.16E-04	4.45E-02	Yes
rs4233701	2	23706216	G/C	*KLHL29*	intronic	4.50E-02	1.15E-04	4.50E-02	Yes
rs4704942	5	158466352	G/C	*RP11*	intergenic	4.95E-02	1.83E-05	4.95E-02	No
rs6016377	20	40544088	C/T	*SNORD112*	intergenic	2.95E-02	1.83E-07	2.95E-02	No
rs630014	9	133274306	A/T	*ABO*	intronic	2.05E-03	9.57E-03	9.57E-03	Yes
rs6673081	1	155017119	T/A	*ZBTB7B*	3'-UTR	3.73E-02	2.11E-07	3.73E-02	Yes
rs6713510	2	226169783	G/C	*LOC646736*	intronic	1.07E-02	1.63E-02	1.63E-02	Yes
rs8039305	15	90879313	T/A	*FURIN*	intronic	2.86E-11	3.92E-07	3.92E-07	Yes
rs866919	10	30224354	C/G	*RP11*	intergenic	3.92E-02	2.45E-02	3.92E-02	Yes
rs965098	21	15185306	G/C	*JCAD*	intergenic	4.84E-02	4.08E-02	4.84E-02	Yes

Supplementary Table 10. 52 SNPs for genetic association from BW to CAD by mendelian randomization analysis.

SNP	Gene	Alt	BW		CAD	
			P	*β*	*P*	*β*
rs1011939	*GPR139*	G/C	3.00E-09	2.36E-02	2.46E-01	-1.20E-02
rs10402712	*PEPD*	A/T	2.00E-08	2.29E-02	5.33E-03	-2.94E-02
rs10830963	*MTNR1B*	G/C	1.00E-07	2.20E-02	5.27E-02	2.04E-02
rs10935733	*CPA3*	T/A	6.00E-10	2.31E-02	8.81E-02	-1.64E-02
rs1101081	*ESR1*	C/G	6.00E-20	3.70E-02	1.45E-02	-2.61E-02
rs11055034	*APOLD1*	C/G	2.00E-08	2.30E-02	8.28E-01	-2.32E-03
rs113086489	*CLDN7*	T/A	1.00E-15	2.96E-02	3.59E-01	-8.98E-03
rs11765649	*IGF2BP3*	T/A	1.00E-09	2.58E-02	4.07E-01	-9.34E-03
rs12543725	*SLC45A4*	G/C	2.00E-09	2.23E-02	9.53E-03	-2.52E-02
rs12823128	*ITPR2*	T/A	3.00E-08	2.04E-02	2.13E-01	1.20E-02
rs12906125	*FES*	G/C	1.00E-08	2.26E-02	8.64E-08	-5.97E-02
rs12942207	*SP6*	C/G	3.00E-09	2.38E-02	9.52E-01	-6.04E-04
rs13266210	*ANK1*	A/T	2.00E-11	2.98E-02	6.57E-01	5.10E-03
rs13322435	*CCNL1*	A/T	1.00E-42	5.24E-02	1.64E-01	-1.37E-02
rs134594	*KREMEN1*	C/G	2.00E-08	2.15E-02	3.07E-02	-2.07E-02
rs1351394	*HMGA2*	T/A	2.00E-33	4.30E-02	3.40E-02	-2.03E-02
rs1374204	*EPAS1*	T/A	2.00E-29	4.59E-02	3.98E-01	-8.52E-03
rs138715366	*YKT6*	C/G	1.00E-26	2.44E-01	1.29E-01	-9.68E-02
rs1415701	*L3MBTL3*	G/C	4.00E-11	2.70E-02	8.78E-02	-1.79E-02
rs144843919	*SUZ12P1*	G/C	2.00E-09	6.85E-02	6.89E-02	-7.51E-02
rs1819436	*RNF219*	C/G	2.00E-09	3.29E-02	3.60E-02	2.96E-02
rs2150052	*LPAR1*	T/A	3.00E-08	2.03E-02	1.75E-01	-1.25E-02
rs2229742	*NRIP1*	G/C	2.00E-08	3.37E-02	1.82E-02	-3.93E-02
rs2242116	*PTH1R*	A/T	1.00E-08	2.09E-02	2.21E-01	1.17E-02
rs2324499	*LINC00332*	G/C	8.00E-09	2.25E-02	8.94E-01	1.39E-03
rs2421016	*PLEKHA1*	T/A	6.00E-09	2.07E-02	1.27E-01	-1.41E-02
rs2473248	*WNT4*	C/G	1.00E-09	3.31E-02	9.76E-01	3.83E-04
rs28510415	*PTCH1*	G/C	4.00E-16	5.26E-02	1.06E-01	-2.74E-02
rs28530618	*C20orf203*	A/T	8.00E-11	2.40E-02	5.89E-01	5.16E-03
rs2854355	*RB1*	G/C	2.00E-08	2.40E-02	2.15E-01	1.33E-02
rs35261542	*CDKAL1*	C/G	1.00E-28	4.44E-02	3.05E-02	-2.21E-02
rs3753639	*ZBTB7B*	C/G	1.00E-12	3.10E-02	6.05E-03	-3.33E-02
rs6016377	*MAFB*	T/A	4.00E-10	2.39E-02	7.13E-03	-2.61E-02
rs6040076	*JAG1*	C/G	7.00E-09	2.18E-02	1.73E-01	1.32E-02
rs61154119	*ACTL9*	T/A	2.00E-08	2.83E-02	6.48E-01	6.01E-03
rs61830764	*DTL*	A/T	5.00E-08	2.18E-02	9.62E-01	5.36E-04
rs61862780	*HHEX*	T/A	1.00E-14	2.79E-02	7.55E-02	-1.69E-02
rs62240962	*SREBF2*	C/G	4.00E-12	4.70E-02	7.52E-01	-6.01E-03
rs62466330	*MLXIPL*	C/G	6.00E-12	5.12E-02	9.99E-02	-3.68E-02
rs6537307	*HHIP*	G/C	1.00E-12	2.59E-02	9.11E-01	-1.05E-03
rs6989280	*TRIB1*	G/C	5.00E-08	2.21E-02	5.61E-01	-5.95E-03
rs700059	*STRBP*	G/C	1.00E-12	3.61E-02	7.39E-01	4.23E-03
rs7076938	*ADRB1*	T/A	5.00E-18	3.49E-02	3.97E-01	8.89E-03
rs72480273	*FCGR2B*	C/G	2.00E-09	3.00E-02	2.36E-01	-1.70E-02
rs72851023	*INS*	T/A	7.00E-10	4.63E-02	4.75E-01	1.51E-02

rs7402982	*IGF1R*	A/T	1.00E-09	2.31E-02	5.19E-01	-6.29E-03
rs74233809	*NT5C2*	T/A	2.00E-09	-3.87E-02	4.09E-07	7.21E-02
rs7575873	*ATAD2B*	A/T	6.00E-11	3.62E-02	7.08E-03	-3.87E-02
rs7729301	*EBF1*	A/T	1.00E-09	2.46E-02	1.12E-02	-2.59E-02
rs7742369	*HMGA1*	G/C	1.00E-08	2.68E-02	7.87E-01	-3.53E-03
rs7964361	*IGF1*	A/T	1.00E-08	3.78E-02	2.12E-01	-2.08E-02
rs798489	*GNA12*	C/G	5.00E-09	2.40E-02	9.54E-01	-6.78E-04
rs854037	*Intergenic*	A/T	3.00E-08	2.51E-02	6.33E-01	5.40E-03
rs925098	*LCORL*	G/C	1.00E-15	3.22E-02	1.01E-02	-2.72E-02

Supplementary Table 11. Heterogeneity test assesses whether heterogeneity exists in SNPs.

**Method**	***Q***	***Q*_df**	***P*_value**
Fixed effects meta-analysis (simple SE)	121.202	51	0.1804443
Random effects meta-analysis (delta method)	121.202	51	0.1804443
Maximum likelihood	120.8839	50	0.1289658
MR Egger	121.202	51	0.1143716
Inverse variance weighted	119.6123	51	0.1415539

Supplementary Table 12. Causal association from CAD to BW by mendelian randomization analysis.

**Method**	**nSNP**	***β*(95%CI)**	***P*_value**
Simple median	39	-0.0002 (-0.0152, 0.0149)	0.98
Weighted median	39	-0.0002 (-0.0253, 0.0250)	0.99
Weighted mode	39	0.0163 (-0.0077, 0.0403)	0.19
Maximum likelihood	39	-0.0002 (-0.0155, 0.0152)	0.98
MR Egger	39	0.0162 (-0.0443, 0.0767)	0.60
Inverse variance weighted	39	-0.0002 (-0.0253, 0.0250)	0.99
